# Identification of Ginsentide-like Peptides from *Cacao* Beans with Oxidative Stress Protection

**DOI:** 10.1021/acsomega.5c01401

**Published:** 2025-07-15

**Authors:** Shining Loo, Antony Kam, Stephanie V. Tay, James P. Tam

**Affiliations:** ‡ Wisdom Lake Academy of Pharmacy, Xi’an Jiaotong-Liverpool University, Wuzhong No.111, Renai Road, Suzhou, Jiangsu 215123, People’s Republic of China; § Department of Biosciences and bioinformatics, Xi’an Jiaotong-Liverpool University, Wuzhong No.111, Renai Road, Suzhou, Jiangsu 215123, People’s Republic of China; † School of Biological Sciences, 54761 Nanyang Technological University, Singapore 637551, Singapore

## Abstract

Chocolate, derived from *Theobroma cacao* beans, is valued for its antioxidative benefits. However, little is known about cacao-derived peptides that counter oxidative stress. Ginsentides found in *Panax ginseng* are cysteine-rich peptides that exhibit the “cure-all” health benefits of ginseng by coordinating multiple physiological systems to reduce cellular stress and damage. Here, we report the discovery and characterization of ginsentide-like peptides from *T. cacao* beans and chocolate. By combining database searches, isolation, and mass spectrometry analysis, we identified a panel of 33-residue ginsentide-like peptides, termed cocotides tC1–tC12, which are also rich in methionine. We isolated, purified, sequenced, and chemically replicated cocotide tC1 for detailed characterization. We showed that the highly compact tC1 is resistant to proteolytic degradation, and its fluoresence-labeled form shows cell-penetrating property. Modeling by AlphaFold3 revealed that cocotide tC1 and ginsentide share a similar structural fold, but tC1 contains a metal-ion-binding site composed of Met and Asn. We used N-terminal biotinylated tC1 and affinity-enrichment mass spectrometry to identify ferrochelatase as one of its intracellular targets. Functional studies demonstrated that cocotide tC1 is a Fe^3+^-binding peptide and a strong antioxidant. Tandem-mass-tag-based quantitative proteomics analysis showed that cocotide tC1 affects proteins associated with mitochondrial functions, oxidative stress, cell proliferation and repair, protein ubiquitination, and DNA damage in HaCaT keratinocytes. Altogether, our findings indicate that cocotides are ginsentide-like peptides with the potential to maintain cellular homeostasis and protect against oxidative damage in skin keratinocytes.

## Graphical abstract

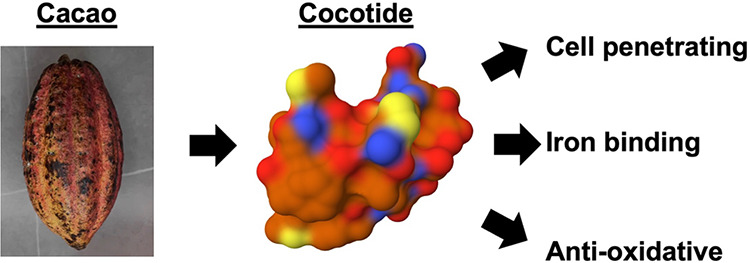

## Introduction

*Theobroma cacao*, of the Malvaceae family, has been cultivated and consumed for millennia. It is valued not only for its flavor but also for its medicinal properties. Dark chocolate, derived from cacao beans, is recognized as a “superfood” due to its health benefits, which include mitigating inflammatory diseases such as diabetes, hypertension, oxidative stress, and cardiovascular conditions.^1,2^ Thus far, studies on cacao-derived bioactive compounds have primarily focused on lipids and secondary metabolites for their antioxidant properties. Notably, these compounds include polyphenols such as flavanols, proanthocyanidins, and anthocyanins.^2^ They are believed to mediate cacao’s anti-inflammatory and cardioprotective effects. Additionally, cacao is a rich source of minerals like magnesium, potassium, copper, and iron, which contribute to its cardiovascular benefits.^3^ Thus far, there has been no systematic study on the identification of bioactive peptides, particularly metabolically stable, proteolysis-resistant cysteine-rich peptides (CRPs) in cacao beans with potential medicinal benefits.

Plant-derived CRPs that contain six to ten cysteine residues and fewer than 50 amino acids in length are regarded as small proteins. They exhibit high resistance to proteolytic degradation, tolerance to high temperature and acidic degradation.^4–7^ CRPs are classified into different families based on their cysteine motifs, sequence homology, and tertiary structural folds.^8^ The major CRP families in plants include thionins, defensins, chitin- and nonchitin-binding hevein-like peptides, and knottin-type peptides, which can be linear or cyclic.^8^

Recently, our laboratory discovered a new family of CRPs termed ginsentides.^9–12^ Ginsentides are found abundantly in *Panax ginseng*, with *Panax quinquefolius* and *Panax notoginseng* as notable representatives. Both are highly popular medicinal herbs used worldwide to treat various ailments. Based on their cysteine motif and disulfide connectivity of Cys I–IV, Cys II–VI, Cys III–VII, and Cys V–VIII, ginsentides belong to the superfamily of 8-cysteine nonchitin-binding hevein-like peptides.^9^ In addition to being cysteine-rich, they are also Gly-rich with nine glycine. Together, their Cys and Gly account for 17 residues in the 31-amino-acid ginsentide TP1, the prototype of the ginsentide family.^9^ Also, ginsentide structures are pseudocyclic, with terminal cysteine residues forming disulfide bonds that connect to the internal peptide backbone, which contributes to their compact structure and high stability against various forms of degradation. Such exceptional resistance to proteolysis also makes ginsentide TP1 orally bioavailable.^9,11^

Another appealing property of ginsentides is their ability to penetrate cells and effectively target intracellular proteins.^10,11^ Intracellular proteins are underexplored drug targets, and the large surface area of CRPs such as ginsentides can be advantageous over small-molecule natural products, which often prove too small to inhibit intracellular protein–protein interactions.^13^ *In vitro* and *in vivo* studies have shown that ginsentides are anti-inflammatory, antioxidative, neuroprotective, and cardioprotective, which reduces cellular and physiological stress.^10,11^ Because stress is an underlying cause of chronic diseases such as cardiovascular disorders, metabolic syndrome, and neurodegenerative conditions, ginsentides and ginsentide-like peptides hold promising therapeutic potential. Because of their ability to elicit a broad spectrum of bioactivities, we have proposed that ginsentides are among principal compounds contributing to the “cure-all” medicinal effect of ginseng.^11^ The extraordinary structural advantages and broad-spectrum health benefits of ginsentides as potential candidates and templates for biological drug development led our laboratory to explore ginsentide-like CRPs in nonginseng plants. Recently, we reported the identification of a suite of ginsentide-like peptides named coffeetides from *Coffea canephora* and *Coffea liberica*.^14^ Interestingly, coffeetides not only penetrate cells but also bind to metal ions.^14^

Here, we report the discovery and characterization of a new family of ginsentide-like peptides from *T. cacao* beans, which we term cocotides. We chemically synthesized the prototype cocotide tC1. Using structure prediction, computer modeling, affinity-enrichment mass spectrometry, and tandem mass tag (TMT)-based quantitative proteomic profiling, we show that cocotide tC1 protects cells from oxidative stress and damage in HaCaT keratinocytes. Functional studies further confirmed that tC1 binds metals and is an antioxidant. Furthermore, we show that cocotides are present in commercially available chocolate. Together, our discovery and characterization of these novel ginsentide-like peptides enhance our understanding of cacao’s repertoire of bioactive peptides and natural products.

## Materials and Methods

### Materials

All chemicals and solvents, unless otherwise stated, were purchased from Sigma–Aldrich (St. Louis, MO, United States) and Fisher Scientific (Waltham, MA, United States).

### Plant Materials

All plant materials were collected from the Nanyang Community Herb Garden, Nanyang Technological University, Singapore. Authentication was performed by Mr. Paul Leong from the Singapore Botany Center based on macroscopic and microscopic analyses. Voucher samples were deposited at the Nanyang Technological University Herbarium, School of Biological Sciences, Singapore.

### Mass Spectrometry Screening of *Theobroma cacao* Aqueous Extracts

Fresh plant parts of *Theobroma cacao* were extracted with water for 15 min at room temperature in a 1:10 ratio. The aqueous extract was vortexed vigorously, centrifuged at 16,000 × *g* for 5 min at 4 °C, and subjected to flash chromatography by C18 solid phase extraction (SPE) columns (Waters, USA). The fractions were eluted with 60% ethanol/0.01% trifluoroacetic acid (TFA) and analyzed by matrix-assisted laser desorption/ionization time-of-flight mass spectrometry (MALDI-TOF MS) (AB SCIEX 5800 MALDI-TOF/TOF).

### *S*-Reduction and *S*-alkylation

Ten μg portion of the purified peptide was reduced using 10 mM dithiothreitol (DTT) and incubated for 2 h at 37 °C in 100 mM ammonium bicarbonate buffer (pH 8.0). The *S*-reduced peptides were then subjected to *S*-alkylation with 60 mM iodoacetamide (IAM) at 37 °C for 45 min. The *S*-alkylated peptide was subjected to MALDI-TOF MS, and the observed corresponding mass shift was used to determine the number of cysteine residues found in the peptide sequence.

### Primary Sequence Determination Using Trapped Ion Mobility spectrometry-Time of Flight Tandem Mass Spectrometry

The primary sequences of cocotides from *Theobroma cacao* aqueous extracts were determined by LC-MS/MS sequencing as described previously.^7^ The C18-SPE fractionated *Theobroma cacao* aqueous extracts were redissolved in 20 mM DTT at 37 °C for 1 h followed by *S*-alkylation with 200 mM iodoacetamide at 37 °C for 1 h. The mixture was desalted with a C18 Zip-tip and subjected to analysis on a trapped ion mobility spectrometer-time-of-flight tandem mass spectrometer (tims-TOF MS/MS). Data analysis was performed using PEAKS Studio (version 11, Bioinformatics Solutions, Waterloo, ON, Canada) with a precursor ion tolerance of 10 ppm and a fragment ion tolerance of 0.05 Da. Carbamidomethylation at Cys was set as a fixed modification. Deamidation of Asp and Glu, oxidation of Met, and acetylation at Lys and N-term were set as variable modification. Peptide sequencing was performed using PEAKS DB protein identification, which integrates database search of an in-house ginsentide-like peptide library derived from *Theobroma cacao* transcriptome with *de novo* sequencing.

### Structural Modeling by Alphafold3

The three-dimensional structures of cocotide tC1 were predicted using the AlphaFold3 Web server^15^ in two configurations: the apo-structure (using default parameters) and the Fe^3+^-complexed form (with metal ion parametrization enabled). Structural comparisons with ginsentide TP1 (PDB: 2ML7) and coffeetide cC1a (PDB: 6JI7) were conducted through PyMOL-based alignment, which provided root-mean-square deviation (RMSD) values to calculate structural similarity.

### Peptide Stability Assay

Stability studies were conducted using purified cocotide tC1 (0.1 M) and *S*-alkylated tC1 (iodoacetamido) under specific buffer conditions. Pepsin stability was assessed by incubating the samples in a mixture of pepsin at a ratio of 50:1 (w/v) in 0.2 M HCl at 37 °C. For trypsin stability, the samples were treated with trypsin in 0.1 M ammonium bicarbonate buffer (pH 8) at 37 °C. Analysis of stability assays involved collecting samples at various time points, which were then analyzed by reversed-phase high-performance liquid chromatography (RP-HPLC) using a linear gradient of mobile phase A (0.05% trifluoroacetic acid in water) and mobile phase B (0.05% trifluoroacetic acid in acetonitrile) on an Aeris Peptide XB-C18 column (Phenomenex). The stability results were expressed as a percentage of the initial concentration and determined by the peak area of the HPLC profile.

### Solid-Phase Peptide Synthesis and Oxidative Folding of Cocotide tC1 and Its Chemical Probes

Cocotide tC1 was synthesized by utilizing Fmoc-based solid-phase peptide synthesis on chlorotrityl (Cl-MPA) ProTide resin (LL) with the assistance of an automated microwave-assisted peptide synthesizer. The linear precursor peptide was cleaved using a solution containing 92.5% trifluoroacetic acid (TFA), 2.5% water, 2.5% 1,2-ethanedithiol, and 2.5% triisopropylsilane at room temperature for 2 h, followed by precipitation with diethyl ether. The crude cleavage product was then subjected to folding in a mixture comprising 10% dimethyl sulfoxide (DMSO), 90% 0.1 M ammonium bicarbonate (NH_4_HCO_3_) at pH 8, cystamine (10 equiv), and cysteamine (100 equiv) for 1 h at 4 °C. The folded cocotide tC1 was subsequently purified using preparative high-performance liquid chromatography (HPLC) employing a Phenomenex column (250 × 21 mm, 5 μm) and a linear gradient of mobile phase A (0.1% trifluoroacetic acid in water) and mobile phase B (0.1% trifluoroacetic acid in acetonitrile). The folded synthetic tC1, along with FAM-tC1 and folded biotin-tC1, was confirmed through MALDI-TOF MS analysis. Additionally, reversed-phase HPLC was conducted to compare the physical characteristics of synthetic cocotide tC1 with those of its native counterpart.

### Iron-Binding Assay

The High-Select Fe-NTA Phosphopeptide Enrichment Kit (Thermo Fisher Scientific, USA) was used to investigate the iron-binding abilities of cocotide tC1. Briefly, cocotide tC1 was suspended in the binding buffer provided. The spin column was first equilibrated by adding 200 μL of the binding buffer and then centrifuged at 1000 × *g* for 30 s (Thermo Fisher Scientific, USA). The suspended peptide sample was added to the spin column and incubated for 1 h at room temperature. The sample was then centrifuged again at 1000 × *g* for 30 s, and the flowthrough was collected. The spin column was then washed six times with 100 μL of deionized water. Each time, the sample was centrifuged at 1000 × *g* for 30 s before collecting the flowthrough. Finally, 100 μL of the elution buffer provided was added, and the sample was centrifuged at 1000 × *g* for another 30 s before collecting the final eluted flowthrough. All eight flowthroughs were analyzed by using RP-HPLC.

### Ferric Reducing Antioxidant Power Assay

OxiSelect Ferric Reducing Antioxidant Power (FRAP) Assay Kit (Cell Biolabs, USA) was used to measure the antioxidant potential of cocotide tC1. The assay was performed according to the manufacturer’s instructions .

### Cell Cultures

HaCaT (human keratinocyte) cells were cultured in Dulbecco’s modification of Eagle’s medium (DMEM) supplemented with 10% fetal bovine serum and 100 U/mL penicillin and streptomycin. They were grown in a 5% CO2 humidified incubator at 37 °C

### Confocal Microscopy Analysis

To examine the internalization of FAM-tC1 in living cells, the cells were seeded on an 8-well chamber slide (Ibidi). Prior to incubation with FAM-tC1, the cells were stained with Hoechst 333241. FAM-tC1 was incubated on cells in phenol red-free and serum-free medium for 1 h at 37 °C. The slides were washed gently with PBS three times, and the medium was replaced prior to imaging. The slides were observed by using a Zeiss LSM 980 confocal microscope.

### Affinity-Enrichment Mass Spectrometry Profiling

Affinity-enrichment mass spectrometry profiling was performed using immobilized biotin-tC1 on NeutrAvidin UltraLink Resin (Thermo Fisher Scientific, USA). Briefly, 20 μg of HaCaT cell lysate was then added to each tube. The mixture was incubated overnight at 4 °C with end-to-end rotation. The resins were then transferred to Pierce Spin columns (Thermo Fisher Scientific, USA) and washed with PBS 10 times. 50 μL of 0.1 M ammonium bicarbonate buffer (pH 8.5) was added to each of the spin columns. The spin columns were then incubated at 95 °C for 15 min, *S*-reduced using DTT at 65 °C for 1 h, and *S*-alkylated IAM and incubated at 37 °C in a dark room for 1 h. The samples were then subjected to in-solution digestion with LysC and trypsin overnight at room temperature. The samples were then subjected to high-pH C18 flash chromatography using increasing concentrations of acetonitrile at 10%, 20%, 30%, and 50%, supplemented with 0.01% triethylamine. The respective elution tubes were then vacuum-dried.

The resuspended samples were then analyzed using LC-MS/MS. A Dionex UltiMate 3000 ultrahigh-performance liquid chromatography (UHPLC) system coupled with an Orbitrap Elite mass spectrometer (Thermo Fisher Scientific, USA) was used to perform liquid chromatography with tandem mass spectrometry (LC-MS/MS). The enzymatically digested fragments were first dissolved in 0.1% formic acid solution and sonicated for 10 min using an SB-120DT ultrasonic cleaner (RS Components, Singapore). The mixture was then separated by an Acclaim PepMap RSL column (Thermo Fisher Scientific, USA) with a linear gradient of mobile phase A (0.1% formic acid in LC-MS grade H_2_O) and mobile phase B (0.1% formic acid in LC-MS grade ACN). A Microm Thermo Captive Spray nanoelectrospray ion source (Bruker–Michrom, USA) was used to spray the samples into the mass spectrometer with a source voltage of 1.5 kV. The MS scan range was set at 350–1600 *m*/*z* with a resolution of 60,000 at 400 *m*/*z*. Additionally, the Fourier transform-MS/MS scan range was set at 150–2000 *m*/*z*, with a resolution of 15,000 at 400 *m*/*z*. For the threshold, the 10 most intense ions with at least 500 counts were selected for high-energy collision dissociation fragmentation. The maximum ion accumulation time was set at 120 ms, and the fragmentation was performed using 32% normalized collision energy.

Proteomic analysis was performed using the PEAKS Studio 11 software tool with a tolerance of 10 ppm for MS and 0.05 Da for MS/MS. The UniProt human proteome (Proteome ID: UP000005640) was used as the reference proteome. Carbamidomethyl at cysteine was set as a fixed modification and methionine oxidation was set as a variable modification. Digestion by trypsin with a maximum of 2 missed cleavages was defined for the search parameters. Quantification was performed using label-free quantification by PEAKS Studio 11. A volcano plot was generated to identify differential interacting proteins that were enriched by at least 2-fold in the experimental group with significance value >15. The complete data set (three biological replicates each of blank controls and biotin-tC1 samples, four fractions per sample) is publicly accessible through the ProteomeXchange Consortium via the JPOST repository (accession: PXD062883).^16^

### Tandem-Mass Tag Quantitative Proteomic Profiling

Tandem-mass tag quantitative proteomic profiling was performed according to the manufacturer’s instructions . Briefly, HaCaT cells treated with or without cocotide tC1 were harvested and lysed by sonication for 3 min at 25% amplitude with a 2-s-on, 3-s-off pulse time in 8 M urea and 10 mM ammonium bicarbonate supplemented with a protease inhibitor. After centrifugation, quantification of the total proteins was performed by the BCA method. A total of 100 μg of protein from each condition was subjected to in-solution digestion before labeling the resultant peptides using the TMT-10plex Isobaric Label Reagent Set (Thermo Scientific, Rockford, IL, USA) according to the manufacturer’s protocol. The labeled samples were combined prior to high pH fractionation on an Xbridge C18 column (4.6 × 250 mm, Waters, Milford, MA, USA) and subsequent analysis by LC-MS/MS. Chromatographic separation was conducted at 1 mL/min using a 60 min gradient profile: 0–5% mobile phase B (ammonium hydroxide in ACN, 0 mmol/L) for 3 min, followed by 5–35% B for 40 min, 35–70% B for 12 min, and 70–100% B for 5 min. Eluate was collected in 1 min fractions and strategically pooled via concatenation based on absorbance measurements at a wavelength of 280 nm. The pooled fractions were then completely dried in the vacuum concentrator, followed by reconstitution in mobile phase A (3% ACN and 0.1% FA) prior to LC-MS/MS analysis.

The fractionated peptides were separated and analyzed using a Dionex Ultimate 3000 RSLCnano system coupled to a Q Exactive instrument (Thermo Fisher Scientific, MA, USA). Separation was performed on a Dionex EASY-Spray 75 μm × 10 cm column packed with PepMap C18 3 μm, 100 Å (Thermo Fisher Scientific) using solvent A (0.1% formic acid) and solvent B (0.1% formic acid in 100% ACN) at a flow rate of 300 nL/min with a 60 min gradient. Peptides were then analyzed on a Q Exactive apparatus with an EASY nanospray source (Thermo Fisher Scientific) at an electrospray potential of 1.8 kV. A full MS scan (350–1,600 *m*/*z* range) was acquired at a resolution of 70,000 and a maximum ion accumulation time of 100 ms. Dynamic exclusion was set to 30 s. The resolution of the higher energy collisional dissociation (HCD) spectra was set to 350,00. The automatic gain control (AGC) settings for the full MS scan and the MS2 scan were 5E6 and 2E5, respectively. The 10 most intense ions above the 2,000-count threshold were selected for fragmentation in HCD, with a maximum ion accumulation time of 120 ms. An isolation width of 2 *m*/*z* was used for MS2. Single and unassigned charged ions were excluded from MS/MS. For HCD, the normalized collision energy was set to 30%. The underfill ratio was defined as 0.3%. Raw data files were processed, searched, and TMT 10-plex was selected as the quantification method using Peaks Studio 11 with the UniProt human proteome (Proteome ID: UP000005640) as the reference proteome. Carbamidomethyl at cysteine was set as a static modification, and methionine oxidation was used as dynamic modifications. Full trypsin digestion with a maximum of three missed cleavages was set as the digestion parameter. The peptide mass tolerance of ±10 ppm and ±0.02 Da for fragment mass were set as mass tolerance parameters. A volcano plot was generated to identify differential interacting proteins that were enriched by at least 2-fold in the experimental group with significance value >15. The complete data set is publicly accessible through the ProteomeXchange consortium via JPOST repository (JPOST accession: PXD062885).^16^

### Signal Peptide Prediction

The identification of signal peptide cleavage positions was performed through computational analysis utilizing the SignalP 4.1 server (default D-cutoff values).^17^

### Statistics

Statistical comparisons were performed using GraphPad version 8.2.1 (US). Data were analyzed using one-way analysis of variance (ANOVA) followed by Newman–Keuls post hoc tests. Data were expressed as mean ± SD, and *p* < 0.05 was considered statistically significant.

## Results

### Identification and Characterization of Ginsentide-like Peptides from *Theobroma cacao*

In our previous publication on ginsentides,^9^ we searched for similar sequences across NCBI and OneKP databases using TBLASTN and BLASTP. Our search parameters included the distinctive cysteine motif (CX_n_CX_n_CCX_n_CXCX_n_CX_n_C) and the precursor sequence of ginsentide TP1, which led us to identify ginsentide-like sequences in *Theobroma cacao*.^18^ The cysteine motif of ginsentide TP1, CX_n_CX_n_CCX_n_CXCX_n_CX_n_C, is also shared by both chitin-binding and nonchitin binding 8-cysteine hevein-like peptides (HLPs). While chitin-binding 8C-HLPs contain a highly conserved SX­(Φ)­X­(Φ) domain (where Φ represents aromatic residues) in intercysteine loop 3 (CysIV-CysV) and six-residue loop 4 (CysV-CysVI) with a conserved aromatic residue essential for chitin binding, ginsentides lack this chitin-binding domain and instead feature highly shortened one-amino-acid intercysteine loop 4 as a CXC motif.^9^ Thus, we termed these 8-cysteine ginsentide-like HLPs in *T. cacao* as cocotides.

We found 12 distinct cocotides, named tC1–tC12, all sharing precursor arrangements similar to those of ginsentides, comprising an N-terminal signal peptide (identified via SignalP V4.1), a propeptide domain, and a mature cocotide domain (Figure 1, top and Table S1). The mature cocotide domain was characterized through molecular weight determination and mass spectrometry sequencing as detailed in subsequent peptidomic analyses. The amino acid sequences of cocotides tC1–tC12 are highly conserved with 85–90% similarity. Notably, they are exceptionally rich in cysteine and glycine residues: eight cysteine residues and seven glycine residues (Figure 1, bottom). Such a high level of similarity in biosynthetic precursor and sequence conservation of being Cys- and Gly-rich firmly places cocotides in the family of ginsentide-like peptides. Differing from ginsentides, cocotides contain two to three methionine residues.

**Figure 1:**
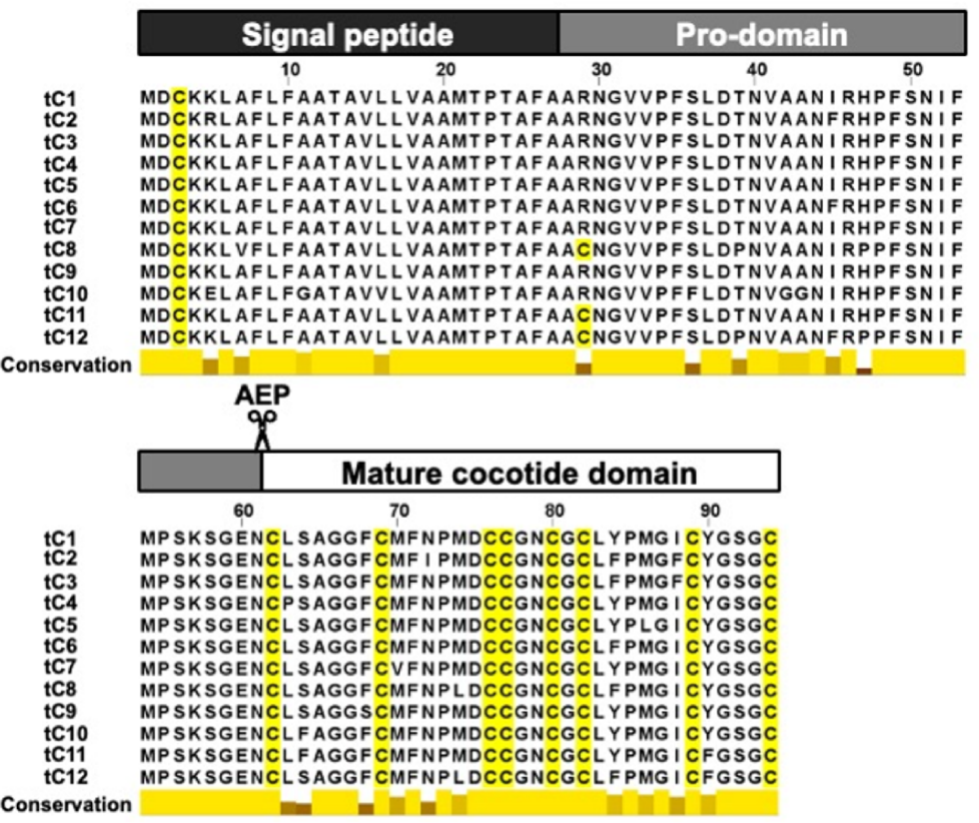
Biosynthesis of cocotides tC1–tC12. Cocotides have three domain precursors: a signal peptide, a prodomain, and a mature cocotide domain which is bioprocessed by asparaginyl endopeptidases (AEP) between Asn61 and Cys62.

To confirm the presence of cocotides in *T. cacao*, we performed matrix-assisted laser desorption/ionization time-of-flight mass spectrometry (MALDI-ToF MS) to profile aqueous extracts derived from various parts of the cacao plant. They include beans, husk, and pulp. The mass spectra of various cacao bean extracts revealed a distinct cluster of peaks in the 2–5 kDa range, with cocotide tC1 being the most abundant (Figure 2). Importantly, we also found cocotide tC1 in the aqueous extract of a commercial dark chocolate powder (Figure S1). To confirm the cysteine content of cocotide tC1, we performed a molecular mass shift experiment involving *S*-reduction with dithiothreitol (DTT) and *S*-alkylation with iodoacetamide (IAM) of cysteine residues, which will increase 56 Da per Cys residue. Our results showed an increase of 464 Da, confirming the presence of eight cysteine residues in cocotide tC1 (Figure 3).

**Figure 2:**
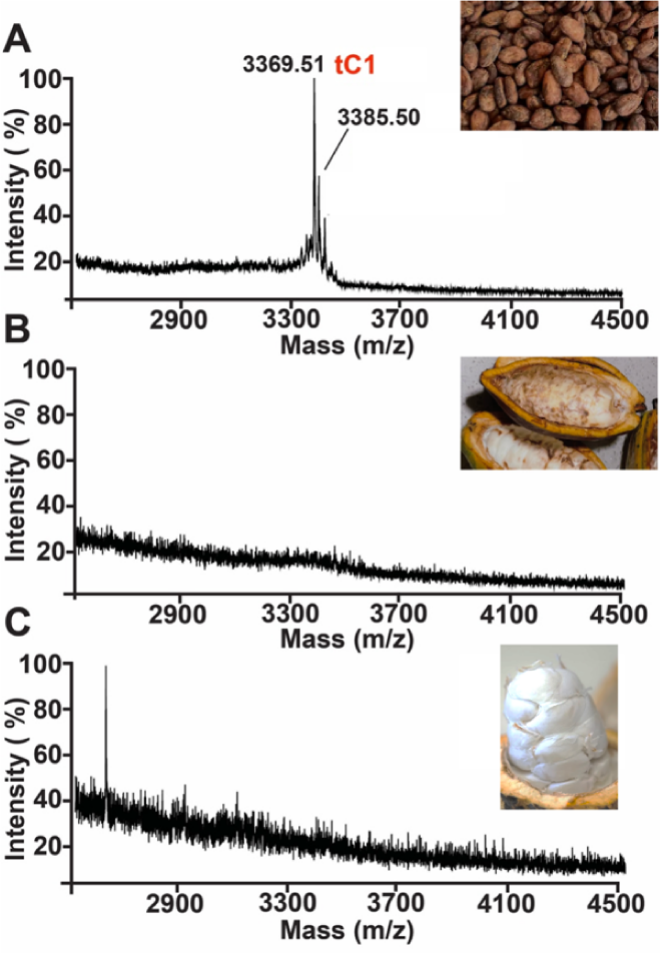
Mass spectrometry profiles of the aqueous extracts from (A) Cacao beans, (B) Cacao husk, and (C) Cacao pulp.

**Figure 3:**
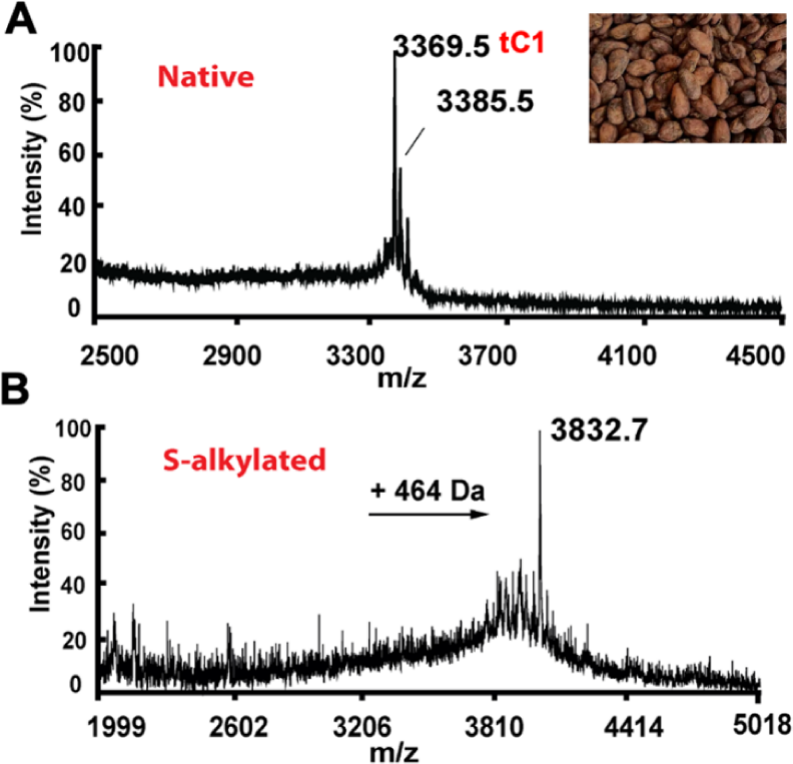
Mass spectrometry profiles of (A) purified cocotide tC1 obtained from cacao beans and (B) *S*-alkylated cocotide tC1.

### Peptidomic Analysis of Aqueous Extracts of *T. cacao* Revealed the Presence of Cocotide tC1, tC2, and tC7

To verify the amino acid sequences of cocotides in *T. cacao*, we performed a peptidomic analysis of its aqueous extracts on the DTT-reduced and IAM-alkylated cocotides reduced, respectively, by DTT and *S*-alkylated by IAM cocotide derivatives using trapped ion mobility spectrometry-time of flight tandem mass spectrometry (tims-TOF MS/MS). Our analysis revealed the presence of three cocotide sequences: tC1, tC2, and tC7 (Figures S2–S4). All three cocotides are highly homologous, with tC2 and tC7 differing from tC1 by two and one amino acid residues, respectively.

### Presence of Met­(O) in Cocotides Resulting in Heterogeneity During Isolation

Profiling by MALDI-ToF-MS also revealed that one of the methionine residues in cocotide tC1 is oxidized to methionine sulfoxide (Met­(O)), as evidenced by an increase of 16 Da in molecular mass. To confirm the presence of Met­(O) in cocotides, we performed post-translational modification analysis on tC1 to examine the susceptibility of different methionine residues to oxidation. We found that 27% of the methionine at position 9 in cocotide tC1 was converted to Met­(O) during extraction and purification, while <5% of Met19 and Met25 were oxidized to Met­(O) (Figure S5). These results suggest that Met19 and Met25 are less solvent exposed compared to Met9. A similar oxidation pattern was seen in cocotide tC2, where Met9 was more prone to oxidation than Met13. In contrast, cocotide tC7, which lacks a methionine residue at position 9, showed minimal oxidation. These findings offer valuable insights into the structural properties and susceptibility of cocotides to post-translational modifications due to varying solvent exposure.

### Sequence and Structural Comparison of Cocotides and Ginsentide TP1

Because cocotide tC1 shares an exceptionally high Cys/Gly composition and Cys motif as ginsentides, it is predicted that they share a common disulfide connectivity (Cys I–IV, Cys II–VI, Cys III–VII, and Cys V–VIII). Unlike neutral ginsentides such as TP1, cocotide tC1 carries a net negative charge. Nevertheless the high sequence similarity and length as well as homology of conserved residues between tC1 and ginsentides permit us to conclude that cocotides and ginsentides would share the same disulfide connectivity. Our conclusion is supported by AlphaFold 3 modeling, which shows that cocotide tC1 maintains disulfide bond arrangements similar to both ginsentide TP1 (PDB: 2ML7, RMSD 1.464) and coffeetide cC1a (PDB: 6JI7, RMSD 1.970), a ginsentide-like peptide isolated from coffee husk (Figure 4). In addition, AlphaFold 3 modeling of the cocotide-metal complex reveals that intercysteine loops 2 (Met13-Asp14 between CysII and CysIII) and 3 (Gly17-Asn18 between CysIV and CysV) of cocotide tC1 form an electronegative pocket comprising Met13 and Asn18, creating a potential binding site for Fe^3+^ ions (Figure 5).

**Figure 4:**
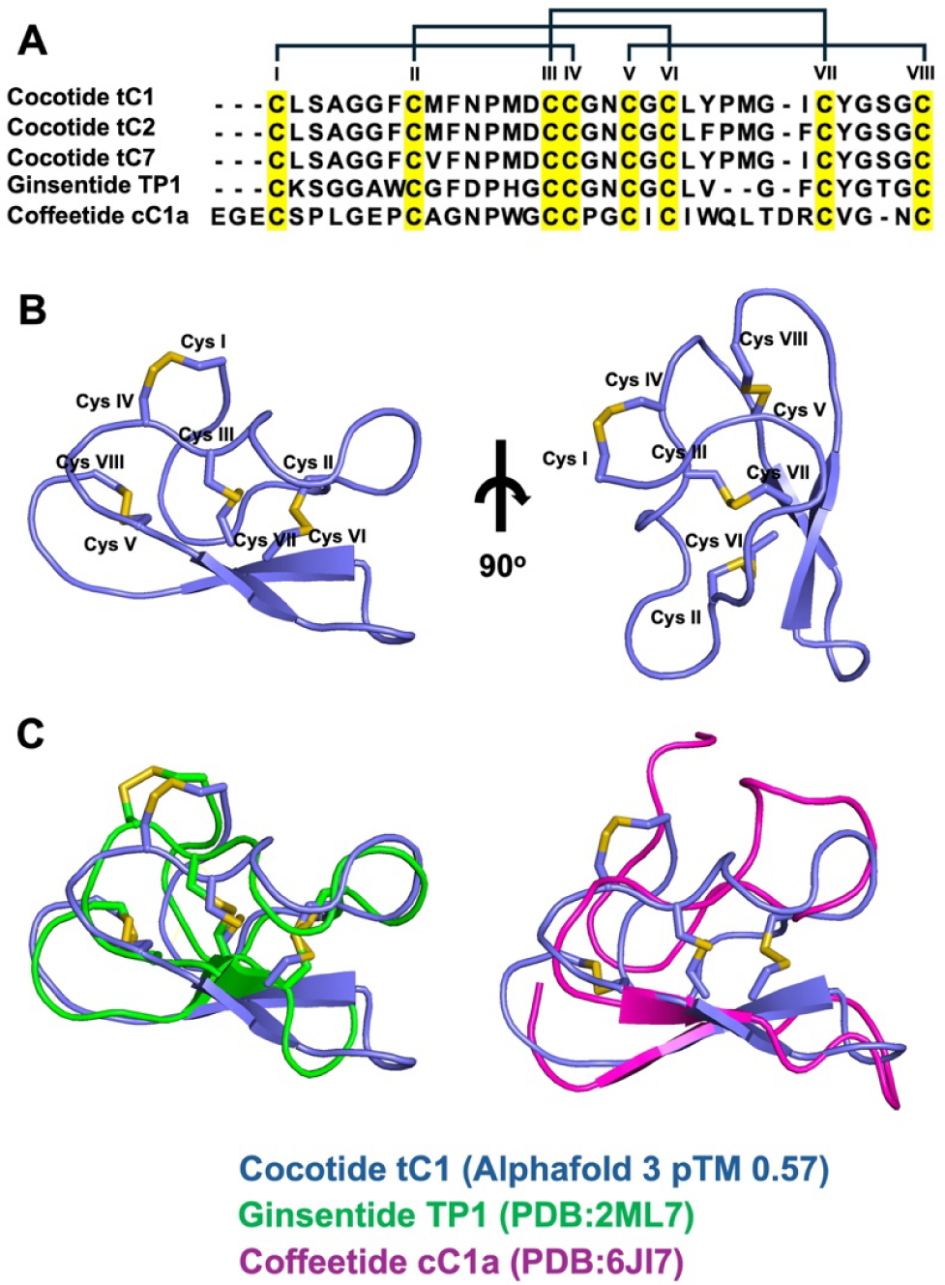
Sequence and structural comparison of cocotide tC1 with related peptides. (A) Sequence alignment of cocotide tC1, tC2, tC7, ginsentide TP1, and coffeetide cC1a. Conserved cysteine residues are highlighted in yellow. Roman numerals indicate the cysteine pairing. (B) Predicted 3D structure of cocotide tC1 modeled by AlphaFold 3. Cysteine residues are labeled and shown in yellow. (C) Structural overlay of the predicted cocotide tC1 structure (blue) with experimentally determined structures of Ginsentide TP1 (green, PDB: 2ML7) and coffeetide cC1a (magenta, PDB: 6JI7). The conserved disulfide bonds are highlighted in yellow.

**Figure 5:**
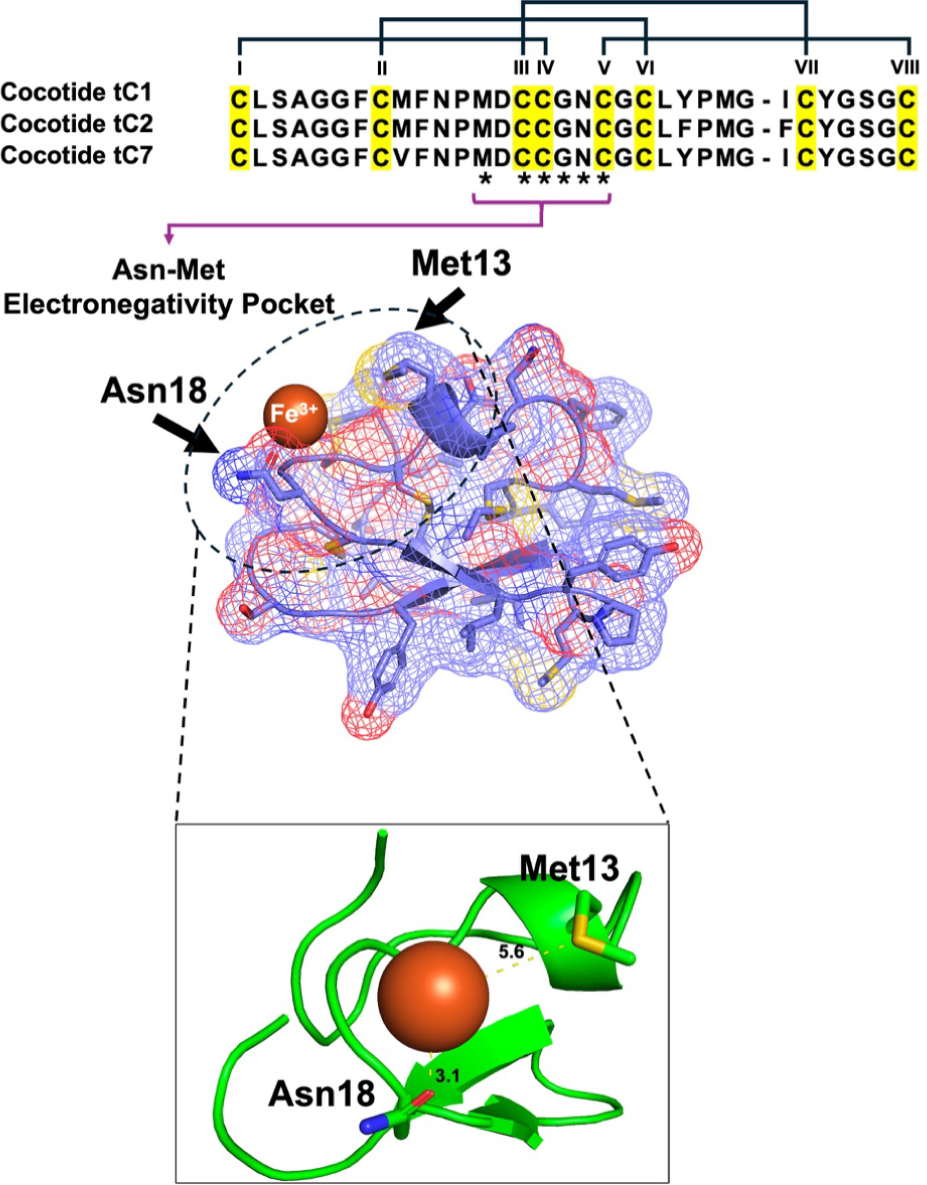
Structural analysis of cocotide variants and their interaction with iron. (Top) Sequence alignment of cocotide tC1, tC2, and tC7, highlighting conserved regions (yellow) and the Met13 residue (asterisks). Roman numerals indicate distinct structural regions. (Bottom) AlphaFold 3 predicted the structure of a representative cocotide, showcasing the Asn–Met electronegativity pocket. Key residues Asn18 and Met13 are labeled. The iron ion (Fe^3+^, orange sphere) is shown interacting with the pocket.

### Chemical Synthesis and Its Chemical Probes to Provide Homogeneous Cocotide tC1

To avoid heterogeneity due to the presence of Met­(O) in cocotides, we chemically synthesized cocotide tC1 to obtain a homogeneous compound for biological assays and functional studies. The synthesized tC1 with an unambiguous identity was then used for all studies described in this paper. In the total synthesis of cocotide tC1, we used an Fmoc-based protecting group scheme together with chlorotrityl (Cl-MPA) resin. The peptide chain assembly was performed by using an automatic microwave-assisted peptide synthesizer. The assembled linear precursor peptide was cleaved using trifluoroacetic acid and a cocktail of scavengers. The crude cleavage product was oxidatively folded in 10% dimethyl sulfoxide (DMSO), 90% 0.1 M NH_4_HCO_3_ (pH 8), cystamine (10 equiv), and cysteamine (100 equiv) for 1 h at 4 °C. The oxidatively folded cocotide tC1 was subjected to preparative HPLC, yielding 70% of the purified product. The purified cocotide tC1 was unambiguously confirmed to its native form using MALDI-ToF MS and coelution with RP-HPLC (Figures 6 and S6–S8).

**Figure 6:**
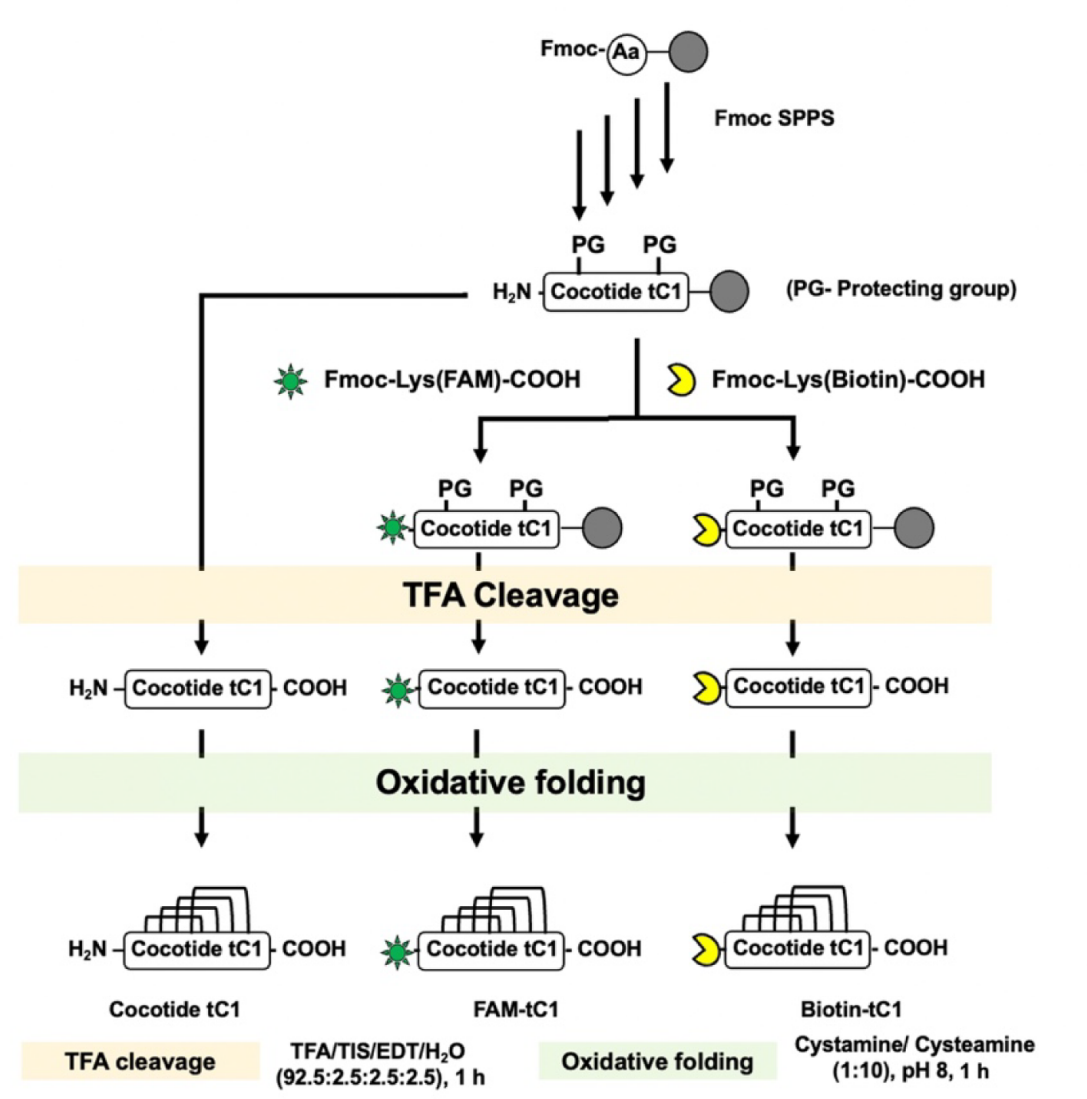
Chemical synthesis of cocotide tC1, fluorescent labeled FAM-tC1, and biotin labeled biotin-tC1 using Fmoc-based solid-phase peptide synthesis. The peptide sequence for FAM-tC1 is K­(FAM)­CLSAGGFCMFNPMDCCGNCGCLYPMGICYGSGC. The peptide sequence for biotin-tC1 is K­(biotin)­CLSAGGFCMFNPMDCCGNCGCLYPMGICYGSGC.

For functional characterizations of cocotide tC1, we prepared various tagged tC1 by chemical synthesis. An additional amino acid, N-terminal Fmoc-Lys (FAM) or Fmoc-Lys­(Biotin), was added to the assembled tC1-peptide resin in the site-specific labeling of cocotide tC1 to maintain a similar overall charge as unlabeled tC1 after its cleavage from the resin support. The Fmoc-Lys­(FAM)- or Fmoc-Lys­(Biotin)-labeled tC1 was deprotected, cleaved, and oxidatively folded as described above. The folded FAM- or Biotin-tC1 was purified by preparative HPLC and identified using MALDI-ToF MS. Figure 6 depicts the chemical synthesis scheme for cocotide tC1 and its site-specifically tagged derivatives.

### Cocotide tC1 is Stable to Proteolytic Degradation

The unique eight-cysteine motif and disulfide connectivity of ginsentide TP1 confer hyperstability against proteolytic degradation. To investigate if cocotide tC1 exhibits similar stability, we performed enzymatic degradation assays using pepsin and trypsin. The *S*-alkylated cocotide tC1, which lacks disulfide bonds, served as a control. Figure 7 illustrates the remarkable stability of cocotide tC1, with over 80% of its structural integrity retained after a 6 h-incubation with either pepsin or trypsin. In contrast, the *S*-alkylated control degraded rapidly. These results indicate that the disulfide scaffold of cocotide tC1 is crucial for its resistance to proteolytic degradation.

**Figure 7:**
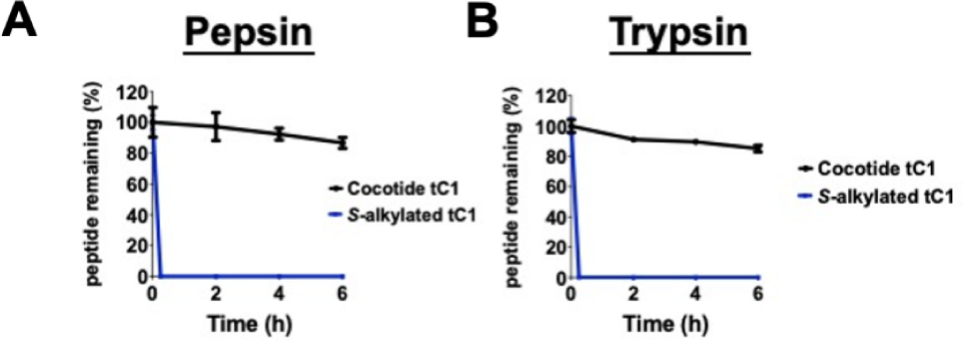
Proteolytic stability assays of cocotide tC1. Cocotide tC1 was incubated with either pepsin or trypsin at 37 °C for up to 6 h. *S-*alkylated tC1 was used as the control. At different time points, samples of cocotide tC1 and the control were quantified using reversed-phase high pressure liquid chromatography (RP-HPLC). The relative peak areas of the RP-HPLC profiles were used to quantify the percentage of peptides remaining after incubation with pepsin or trypsin.

### Cocotide tC1 is a Cell-Penetrating Peptide

Recently, we demonstrated that supercompact plant CRPs, including ginsentides from ginseng, coffeetides from coffee, and roseltides from *Hibiscus sabdariffa*, exhibit cell-penetrating properties.^6,14,19^ To investigate whether cocotide tC1 also translocates into cells, we performed live-cell confocal microscopy in HaCaT keratinocytes using fluorescently labeled FAM-tC1. Our Z-stack images revealed that FAM-tC1 readily penetrates the cells and is found in the cytoplasm following a 1 μM-treatment at 37 °C for 1 h (Figure 8). Notably, while no fluorescent signals were detected in the nucleus, we observed accumulation in specific cellular compartments, the precise identity of which requires further investigation.

**Figure 8:**
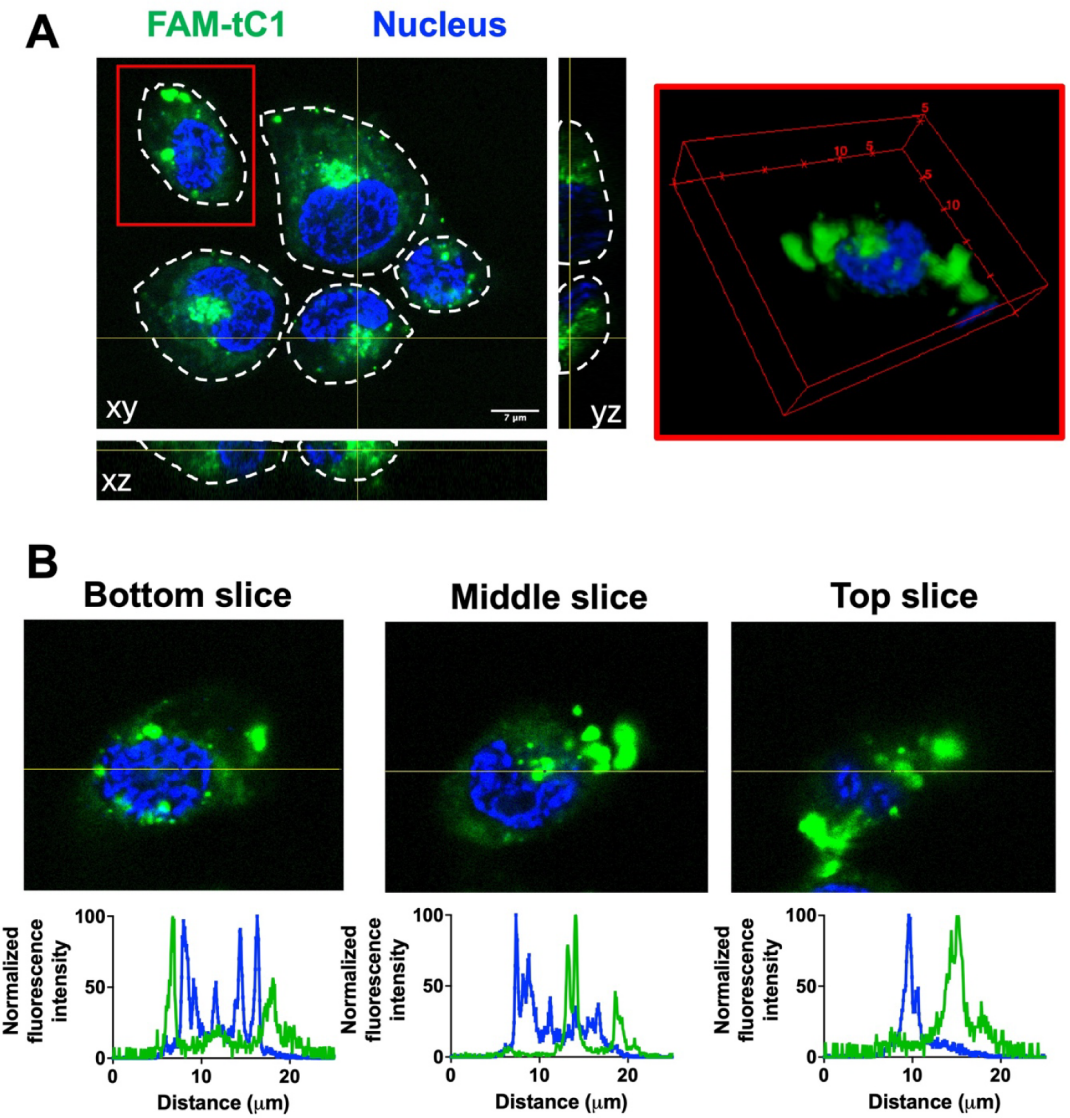
Confocal image of fluorescent labeled FAM-tC1 in HaCaT keratinocytes. (A) 1 μM FAM-tC1 was incubated with HaCaT keratinocytes at 37 °C for 1 h, and Z-stack confocal analysis was performed. Green: FAM-tC1; Blue: Nucleus. The red outline indicates the region of interest (ROI) which is presented as a 3D-rendered Z-stack image on the right. (B) Fluorescence intensity line profiles measured across the ROI at three representative z-depths (bottom, middle, and top slice).

### Affinity-Enrichment Mass Spectrometry

To determine the intracellular targets of cocotide tC1, we used affinity-enriched mass spectrometry to elucidate its potential interacting partners, focusing on metal-interacting proteins. Synthetic biotinylated cocotide tC1 (biotin-tC1) was prepared as described previously and incubated with HaCaT keratinocyte lysates, a well-established keratinocyte cell line for studying skin biology. The complexes were captured using NeutrAvidin streptavidin resin, then denatured, *S*-reduced, *S*-alkylated, and trypsinized into peptide fragments, which were analyzed via LC-MS/MS for sequencing and identification.

Using the Peaks Studio 11 proteomics software, we found a total of 2465 proteins. Of these, 151 proteins showed at least a 2-fold enrichment and a significance value >15 in the pulled-down group compared to the control. After screening against the CRAPome database to exclude common contaminants, 141 proteins were deemed to be significant interacting partners of cocotide tC1 (Figure 9). Among the top five hits, four are metal-binding proteins, including tubulin beta-2B chain (TBB2B), tubulin beta-2A chain (TBB2A), ferrochelatase (HEMH), and glutathione S-transferase (LANC1). Ferrochelatase, an enzyme integral to heme biosynthesis, is particularly noteworthy as it specifically catalyzes the incorporation of ferrous iron (Fe^2+^) into protoporphyrin IX, resulting in heme formation.

**Figure 9:**
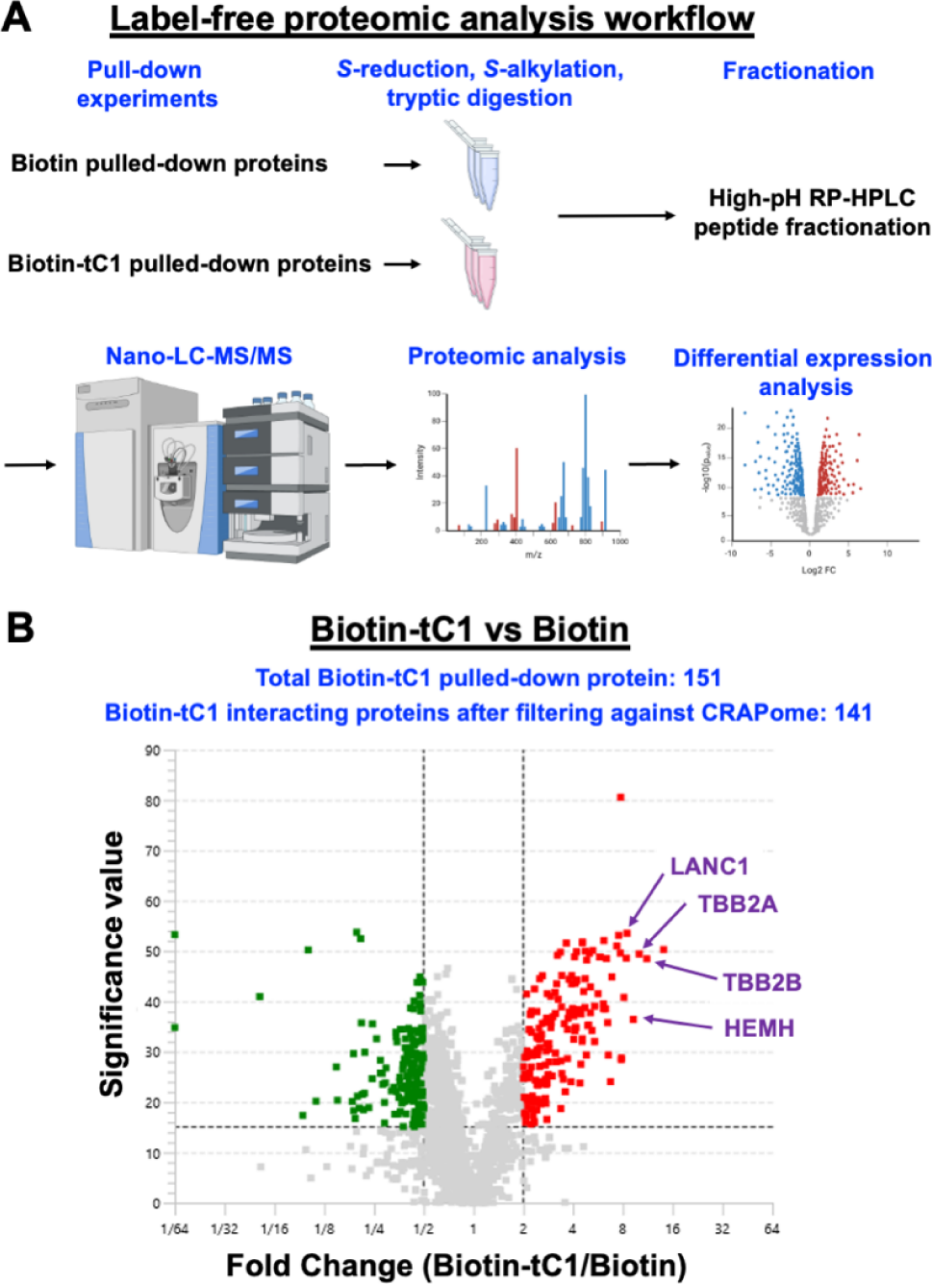
Affinity-enrichment mass spectrometry analysis of biotin-tC1 interacting proteins. (A) Workflow for affinity-enrichment mass spectrometry analysis. The process begins with pull-down experiments using biotin and biotin-tC1, followed by *S*-reduction, *S*-alkylation, and tryptic digestion. Samples then undergo high-pH RP-HPLC peptide fractionation, nano-LC-MS/MS analysis, proteomic analysis, and differential expression analysis. Created in BioRender. Kam, A. (2025) https://BioRender.com/9920pjp. (B) Volcano plot comparing biotin-tC1 versus biotin pull-down results. The *x*-axis represents the fold change (Biotin-tC1/Biotin) on a log 2 scale, while the *y*-axis shows the significance value. A total of 151 proteins were pulled down by biotin-tC1, with 141 proteins remaining after filtering against the CRAPome. Red dots indicate proteins significantly enriched in the biotin-tC1 pull-down, while green dots represent proteins enriched in the Biotin pull-down. Gray dots indicate proteins with no significant difference between the two conditions.

### Cocotide tC1 Interacts with Ferric Ion and is an Antioxidant

AlphaFold 3 modeling suggested that tC1 has an Asn-Met electronegativity pocket (Met13, Asn18), showing potential ferric ion interaction. To examine this, cocotide tC1 was incubated in a spin column with an Fe-NTA agarose resin. After several washes and elutions, RP-HPLC analysis confirmed that cocotide tC1 interacts with the Fe-NTA agarose resin (Figure S9). Additionally, the antioxidant capacity of cocotide tC1 was determined by using the Ferric Reducing Antioxidant Power (FRAP) assay. Our results showed that cocotide tC1 exhibits a FRAP value of 55.1 Fe^2+^ μM/μM, 152-fold greater antioxidant power than ascorbic acid (positive control, 0.36 Fe^2+^ μM/μM).

### Tandem Mass Tag (TMT)-Based Quantitative Proteomic Profiling

To investigate the biological activities of cocotide tC1 on skin, we conducted tandem mass-tag (TMT)-based quantitative proteomics to compare the proteome differences between cocotide tC1-treated and untreated HaCaT cells. HaCaT cells were subjected to overnight serum starvation, followed by treatment with cocotide tC1 for 24 h. The cell lysates were then denatured, *S*-reduced, *S*-alkylated, trypsinized into peptide fragments, labeled with TMT10plex tags, and fractionated for LC-MS/MS analysis (Figure 10). Identified peptide sequences were matched against the human genomic database for differential expression analysis using Peaks Studio 11 software. A total of 8156 proteins were identified. Proteins presenting a minimum 2-fold enrichment and a significance value >15 were considered differentially expressed. Our findings revealed that cocotide tC1 upregulated 19 proteins and downregulated 3 proteins (Figure 10). Notable upregulated proteins include those involved in mitochondrial functions (COX18, KAD7, MICU3), oxidative stress (MT2), cell proliferation and repair (FGF11), protein ubiquitination (UBE3D, RNF26, RNF185), and DNA regulation (H2B1D, H4). These results suggest that cocotide tC1 could play a role in regulating oxidative stress, protein damage, and cell repair/proliferation, potentially linking to wound repair.

**Figure 10:**
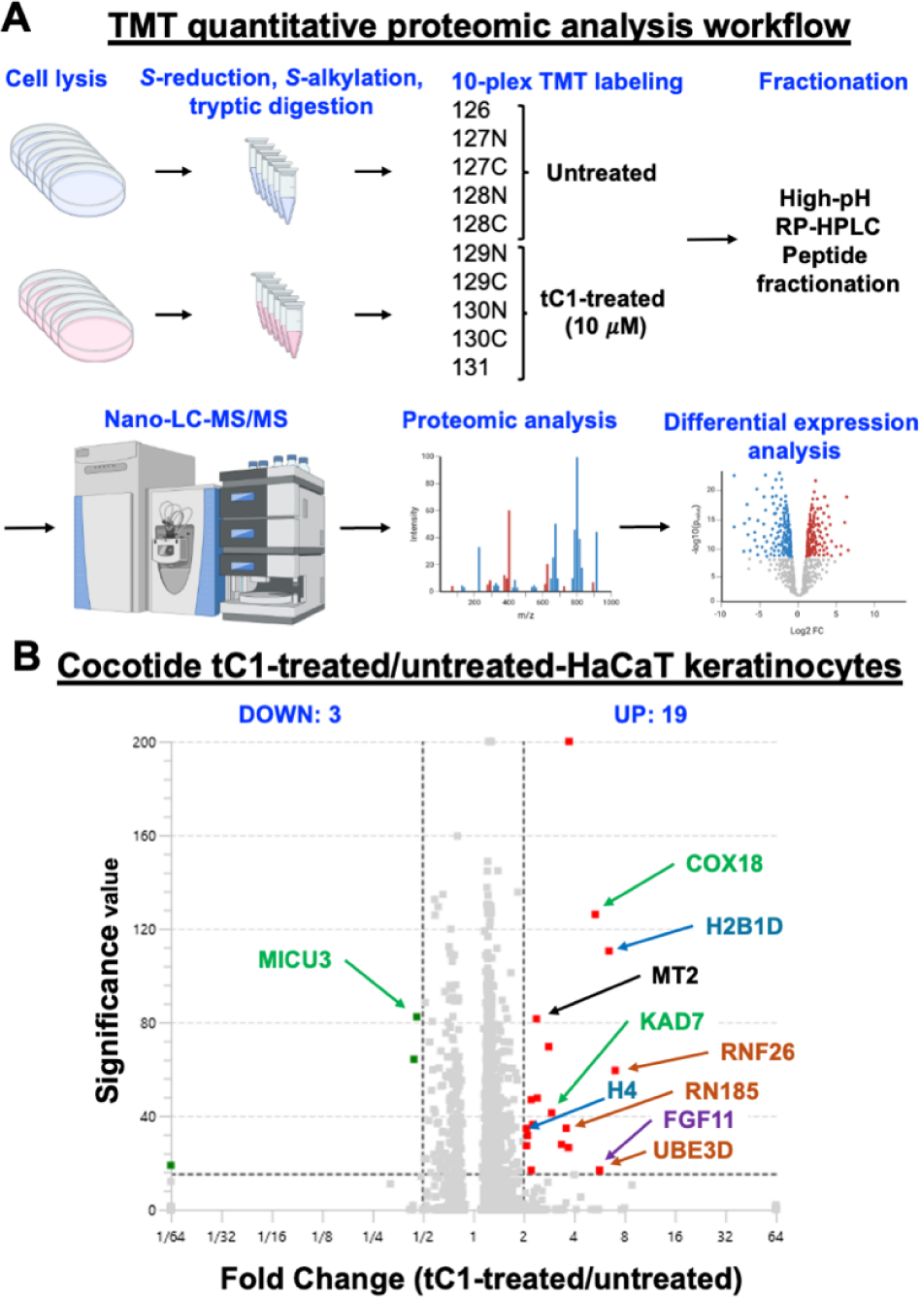
TMT-based quantitative proteomic analysis of tC1-treated HaCaT keratinocytes. (A) Schematic workflow of the TMT quantitative proteomic analysis. HaCaT keratinocytes were treated with or without 10 μM tC1 for 24 h. Cells were lysed, and proteins were *S*-reduced, *S*-alkylated, and digested with trypsin. Peptides were labeled with 10-plex TMT reagents, fractionated by high-pH RP-HPLC, and analyzed by nano-LC-MS/MS. Proteomic data were processed for differential expression analysis. Created in BioRender. Kam, A. (2025) https://BioRender.com/9920pjp. (B) Volcano plot showing differentially expressed proteins in tC1-treated versus untreated HaCaT keratinocytes. The *x*-axis represents the log2 fold change of protein abundance, and the *y*-axis shows the significance value. Upregulated proteins (19) are shown in red, while downregulated proteins (3) are shown in blue.

## Discussion

This work reports the discovery and characterization of cocotides, a novel family of ginsentide-like peptides found in cacao beans and dark chocolate. Our findings reveal the health benefits of cacao beans and provide evidence regarding how chocolate could relieve oxidative stress. In addition, they have the following significance for drug discovery and development of peptide-based drugs. First, they expand the knowledge and structures of bioactive peptidyl natural products from plants regarding their molecular diversity and functions. Second, our discovery impacts the desirable chemical space ranging from 3,000 to 4000 Da for developing cysteine-rich peptides and microproteins, therapeutics that are supercompact, cell-penetrating, and able to target intracellular protein–protein interactions. Currently, they are underrepresented. Third, previous reports on active ingredients contributing to the beneficial health effects of chocolate are limited to small-molecule metabolites and lipids. Our work expands peptide biologics as putative active ingredients. Finally, cocotides have potential for skin repair and wound healing as an antioxidant.

Our interest in ginsentides stems from their broad-spectrum therapeutic effects and health benefits observed in *Panax ginseng*. Ginsentides mitigate stress and protect against cellular damage under various physiological challenges.^10,11^ Encouraged by their robust physicochemical properties and broad-spectrum therapeutic promise, our laboratory investigated other plant sources of ginsentide-like peptides. Recently, we identified coffeetides from coffee plants, which share metal-binding capabilities and structural similarities with ginsentides.^14^ Building on these findings, the current study marks the second report on ginsentide-like peptides outside the *Panax ginseng* family.

The discovery and characterization of plant-derived CRPs generally require a multidisciplinary approach. To identify and characterize cocotides, we used isolation by chromatography, chemical synthesis to obtain enough samples for characterization and functional studies, protein sciences for site-specific modifications, biological assays, informatics for classification and biosynthesis, structural determination, live-cell confocal microscopy, mass spectrometry for peptidomic analysis, affinity enrichment, and TMT-based quantitative proteomic profiling to investigate cocotide interactions with intracellular proteins as well as validation by functional assays.

Using transcriptomic and proteomic methods, we identified 12 distinct cocotides, tC1–tC12. These peptides are highly homologous, consisting of 33 amino acids with molecular weights ranging from 3.3 to 3.4 kDa. They are rich in Cys and Gly residues, accounting for 24% and 21%, respectively, and are characteristic of the ginsentide family. However, cocotides differ from ginsentides by having multiple Met residues. Methionine is sensitive to oxidation and readily converts to its sulfoxide, Met­(O). Indeed, the isolated natural tC1 results in a mixture consisting of multiple forms of both Met and Met­(O). We overcame this challenge of heterogeneity by chemically producing tC1 and its site-labeled derivatives without Met­(O) for characterization and functional studies.

All cocotides possess a CXnCXnCCXnCXCXnCXnC cysteine motif that includes both a tandem connecting CC and CXC motif. Such a motif is shared, in part, by nonchitin-binding hevein-like peptides, 8C-HLP and ginsentides.^8,9,14^ Although experimental 3D structural validation awaits future studies, this motif, along with sequence homology and structural fold, places cocotides firmly in the ginsentide-like family. The extensive disulfide constraints imposed by this motif render cocotides structurally compact and highly resistant to proteolytic degradation like ginsentides and coffeetides. In addition, cocotides are biosynthesized as three-domain precursors comprising an N-terminal signal peptide, a prodomain, and a C-terminal mature peptide.^9,14^ The bioprocessing of precursor proteins to mature cocotides involves at least two proteolytic events. The first cleavage, similar in all three plant families, is achieved by a signal peptidase, which removes the ER signal peptide after the cocotide prodomain. Unlike ginsentides and coffeetides, the second maturation cleavage involves an asparagine endopeptidase (AEP) at the Asn-Cys site to release the mature cocotide domain, as confirmed by peptidomic studies of mature cocotides.

AEPs play a key role in the maturation of seeds and bioprocessing of bioactive peptides and proteins through proteolysis, ligation, and splicing mechanisms.^4,20–23^ Examples of proteolytic type of bioprocessing include the maturation of CRPs such as roseltides from *Hibiscus sabdariffa*, avenatides from oats, and jasmintides from *Jasminum sambac*.^4,7,24^ Biosynthesis of lectins such as ConA and cyclic trypsin inhibitors such as MCoTI occurs through a splicing mechanism, while sunflower seed trypsin inhibitors and cyclotides are processed through end-to-end ligation.^22,23^ In *Theobroma cacao*, the involvement of AEPs not only ensures the proper processing of cocotides but also links their production to the physiological stages of seed development. This connection suggests that cocotides may contribute to seed viability and defense, potentially offering protection against environmental stresses during germination. Understanding the role of AEPs in cocotide biosynthesis could provide valuable insights into the regulation of CRP production and unveils opportunities to harness these enzymes for the synthesis of therapeutic peptides.^21,23,25^

Using fluorescently labeled tC1, we demonstrated that cysteine-rich cocotide tC1 is cell-penetrating. This property is facilitated by clusters of hydrophobic amino acids on the surface of CRPs, which interact with lipid components of the cell membrane, enabling translocation into the cytoplasm.^26^ The dense network of disulfide bonds in tC1 not only provides structural stability but also helps in orienting hydrophobic side chains outward, creating hydrophobic surface patches essential for membrane penetration.^6,19^ Interestingly, negatively charged CRPs can also penetrate cells, as evidenced by our previous studies on roseltides rT1 and rT7.^6,19^ Despite their net negative charge, these peptides utilize hydrophobic surface regions to interact with the cell membrane. Recent examples include coffeetides, which, despite high negative charges, efficiently enter cells due to continuous stretches of apolar amino acids.^14^ This suggests that the overall charge of the peptide does not preclude cell penetration if sufficient hydrophobic interactions are present. The ability of cocotide tC1 and similar peptides to penetrate cells is particularly significant for developing therapeutic peptides and microproteins as intracellular inhibitors. Targeting intracellular proteins remains a considerable challenge, as small-molecule drugs often do not effectively inhibit protein–protein interactions within the cellular environment.^13^ Disulfide-rich, cell-penetrating peptides like ginsentides and ginsentide-like peptides offer a promising alternative. Their structural features allow them to access intracellular spaces and modulate protein functions directly, holding exceptional potential for developing novel biologic drugs to address currently undruggable targets.

Functional divergences of CRPs are influenced by sequence motifs across each intercysteine loop.^5,6,19,27^ Cocotide tC1 features a methionine-aromatic motif (Met9, Phe7, Phe10, Tyr23), which is not found in ginsentides and ginsentide-like CRPs. TIMS-ToF MS/MS post-translational modification analysis revealed that Met9 in tC1 is susceptible to oxidation due to its increased solvent exposure. Despite this susceptibility, the oxidation frequency is only around 27%, possibly due to protective effects from the surrounding aromatic residues.

AlphaFold3 modeling suggests that amino acids in tC1, especially in intercysteine loops 2 and 3, form an electronegative pocket comprising Met13 and Asn18, which potentially facilitates Fe^3+^ binding.^15^ Affinity enrichment mass spectrometry identified ferrochelatase, an enzyme essential for heme biosynthesis with Fe^3+^ binding ability, as a significant interacting partner of tC1. These findings show potential iron-binding sites integral to tC1’s role in iron homeostasis. Given iron’s pivotal role in the Fenton reaction, which generates reactive oxygen species (ROS), tC1’s interaction with iron-related proteins could underlie its protective effects against oxidative stress.^28^ This hypothesis is supported by tC1’s observed interaction with Fe-NTA agarose resin. Additionally, tC1’s antioxidant ability was confirmed through the FRAP assay, demonstrating its free radical scavenging ability. Future studies employing NMR titration experiments are warranted to determine the key amino acid residues contributing to the Fe^3+^ binding properties of cocotide tC1.

Quantitative TMT-proteomics analysis of cocotide tC1-treated HaCaT cells revealed the coordinated upregulation of proteins involved in mitochondrial function, oxidative stress response, and cellular proliferation, though future validation studies are needed to confirm key differentially expressed proteins. Elevated levels of mitochondrial proteins COX18, AK7, and MICU3 suggest enhanced energy metabolism, with MICU3 being particularly important for mitochondrial Ca^2+^ homeostasis and oxidative stress regulation.^29^ The concurrent upregulation of MT2A, a metallothionein family member, provides additional evidence for enhanced cellular protection mechanisms. MT2A functions as a key antioxidant protein through its dual capacity for metal ion sequestration and direct scavenging of reactive oxygen species.^30^ Furthermore, the increased expression of FGF11, a fibroblast growth factor implicated in cellular proliferation pathways,^31^ suggests potential regenerative effects that could be relevant to wound healing applications. The integrated proteomic profile encompassing enhanced mitochondrial function, antioxidative defense, and cell proliferation suggests coordinated skin protective mechanisms. Future functional studies employing wound healing assays, UV-protection experiments, and skin model systems will be essential to validate these effects, elucidate the specific contribution of each protein to cellular phenotypes, and assess the therapeutic potential for dermatological applications.

In conclusion, the discovery of cocotide tC1 from cacao beans advances our understanding of plant-derived peptides with potential therapeutic applications. The unique structural features of tC1 and its cell-penetrating property contribute to its diverse biological functions, including iron binding, antioxidant activities, and modulation of iron homeostasis, which confer protective effects against oxidative damage (Figure 11). The identification of cocotides also provides insight into the molecular basis of chocolate’s reputation as an antioxidant-rich food. The mechanistic pathways of tC1 warrant further elucidation about its role in the health benefits associated with cacao consumption and to explore its potential dermatological applications in wound healing, skin protection, and oxidative stress management.

**Figure 11:**
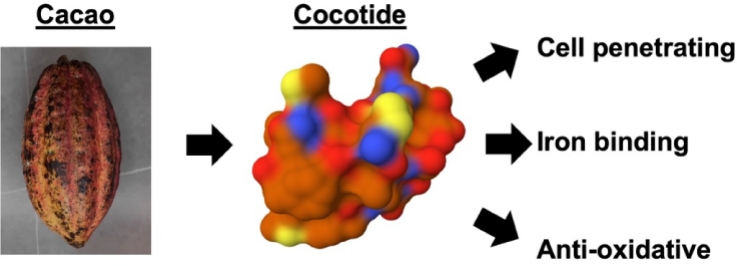
Schematic representation of the identified activities of cocotides.

## Supplementary Material







## Notes

Mass spectrometry profiles of commercial dark chocolate powder, peptide sequencing of cocotides using tims-TOF MS/MS, methionine modification of cocotides, coelution of native and synthetic cocotide tC1 using reversed-phase high performance liquid chromatography, mass spectrometry profile of synthetic cocotide tC1, mass spectrometry profile of Biotin-tC1, and iron-binding properties of cocotide tC1 (PDF)
Protein identification table for TMT-10plex (XLS)
Affinity-enrichment mass spectrometry (XLS)
