# Supplementary material for: Identification of Ginsentide-like Peptides from Cacao Beans with Oxidative Stress Protection
Source: ACS Omega. 2025 Jul 15;10(29):31480–91. doi: 10.1021/acsomega.5c01401 (PMC12311725; doi:10.1021/acsomega.5c01401)
Supplement: Supplementary file 1 [file ao5c01401_si_001.pdf]

Identification of Ginsentide-like peptides from *cacao* beans with oxidative stress protection

Shining Loo[1,3,*,#], Antony Kam[2,3,#], Stephanie V. Tay[3], James P. Tam[3, $]

[1] Wisdom Lake Academy of Pharmacy, Xi'an Jiaotong-Liverpool University, Wuzhong No.111, Renai Road, Suzhou, Jiangsu, 215123, People's Republic of China
[2] Department of Biosciences and bioinformatics, Xi'an Jiaotong-Liverpool University, Wuzhong No.111, Renai Road, Suzhou, Jiangsu, 215123, People's Republic of China
[3] School of Biological Sciences, Nanyang Technological University, Singapore 637551.

[#] These authors contributed equally to this work

Correspondence:

*Dr Shining Loo, Wisdom Lake Academy of Pharmacy, Xi'an Jiaotong-Liverpool University, Wuzhong No.111, Renai Road, Suzhou, Jiangsu, 215123, People's Republic of China. Email: shining.loo@xjtlu.edu.cn

[$]Professor James P. Tam, School of Biological Sciences, Nanyang Technological University, 60 Nanyang Drive, 637551, Singapore. Email: JPTam@ntu.edu.sg

Table S1. Amino acid sequence of cocotides

Peptide	Amino acid sequence	Molecular weight (Da)[1]	Charge[2]
**tC1**	CLSAGGFCMFNPMDCCGNCGCLYPMGICYGSGC	3368.18	-1
**tC2**	CLSAGGFCMFIPMDCCGNCGCLFPMGFCYGSGC	3385.21	-1
**tC3**	CLSAGGFCMFNPMDCCGNCGCLFPMGFCYGSGC	3386.17	-1
**tC4**	CPSAGGFCMFNPMDCCGNCGCLYPMGICYGSGC	3352.15	-1
**tC5**	CLSAGGFCMFNPMDCCGNCGCLYPLGICYGSGC	3350.22	-1
**tC6**	CLSAGGFCMFNPMDCCGNCGCLFPMGICYGSGC	3352.19	-1
**tC7**	CLSAGGFCVFNPMDCCGNCGCLYPMGICYGSGC	3336.21	-1
**tC8**	CLSAGGFCMFNPLDCCGNCGCLFPMGICYGSGC	3334.23	-1
**tC9**	CLSAGGSCMFNPMDCCGNCGCLYPMGICYGSGC	3308.14	-1
**tC10**	CLFAGGFCMFNPMDCCGNCGCLYPMGICYGSGC	3428.22	-1
**tC11**	CLFAGGFCMFNPMDCCGNCGCLYPMGICFGSGC	3412.22	-1
**tC12**	CLSAGGFCMFNPLDCCGNCGCLFPMGICFGSGC	3318.23	-1

Supplementary figures

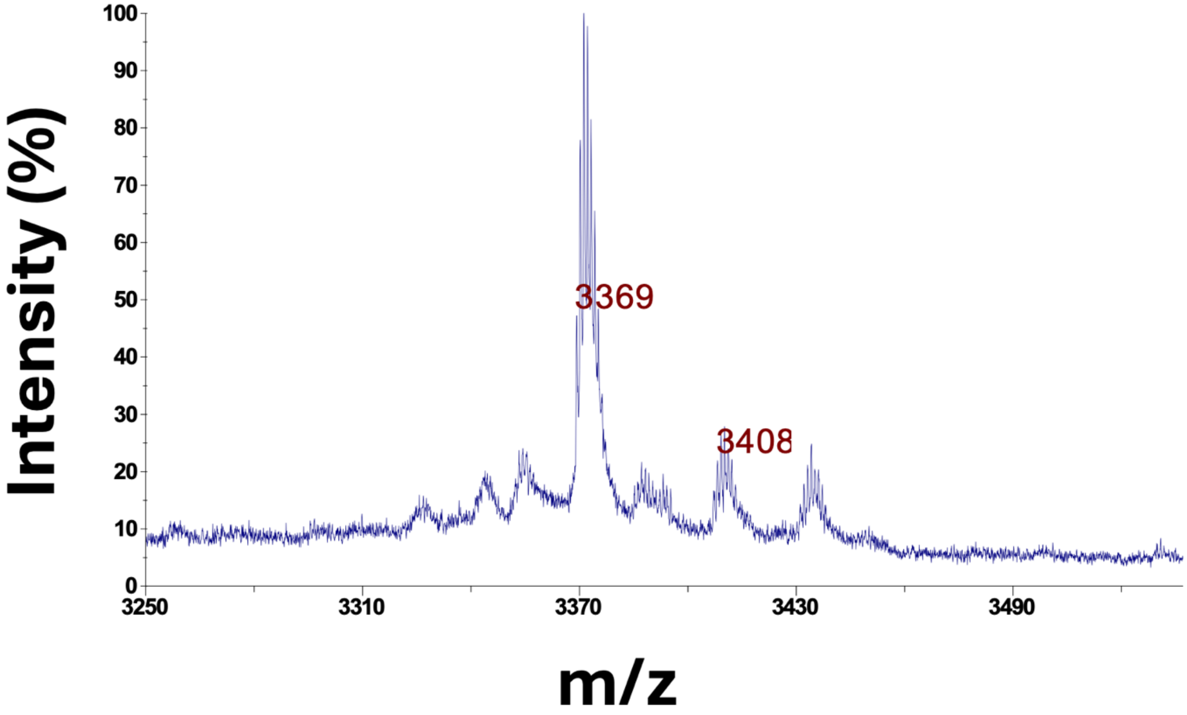

Figure S1. Mass spectrometry profiles of the aqueous extracts from commercial dark chocolate powder.

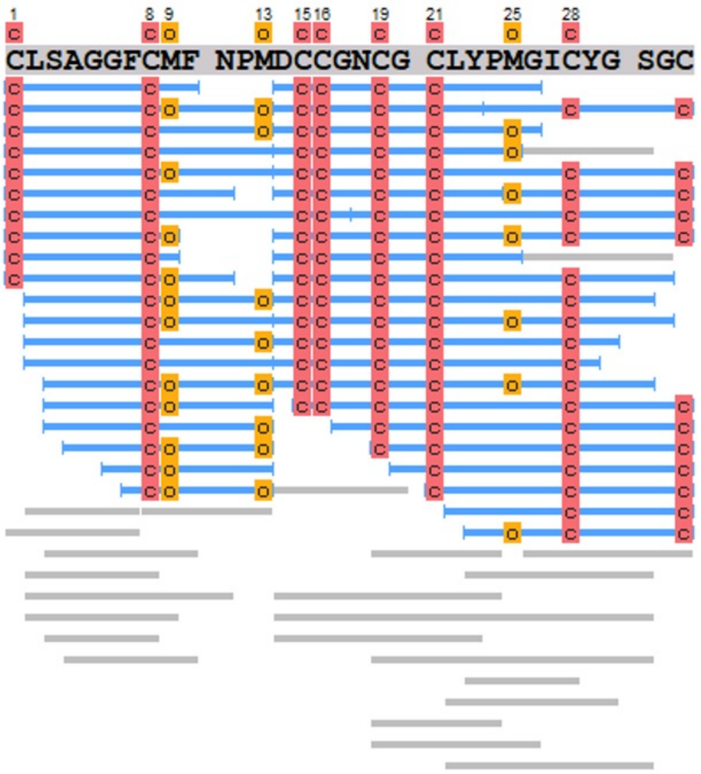

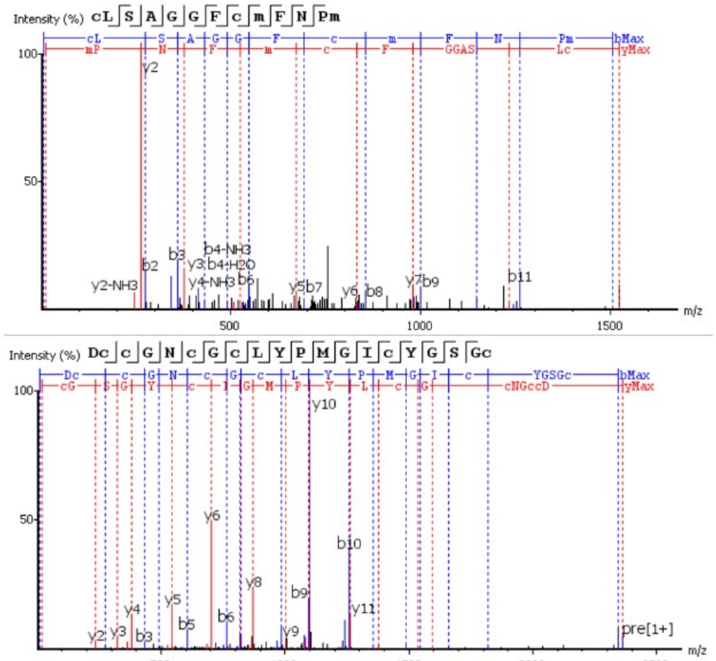

Figure S2. Peptide sequencing of cocotide tC1 using tims-TOF MS/MS

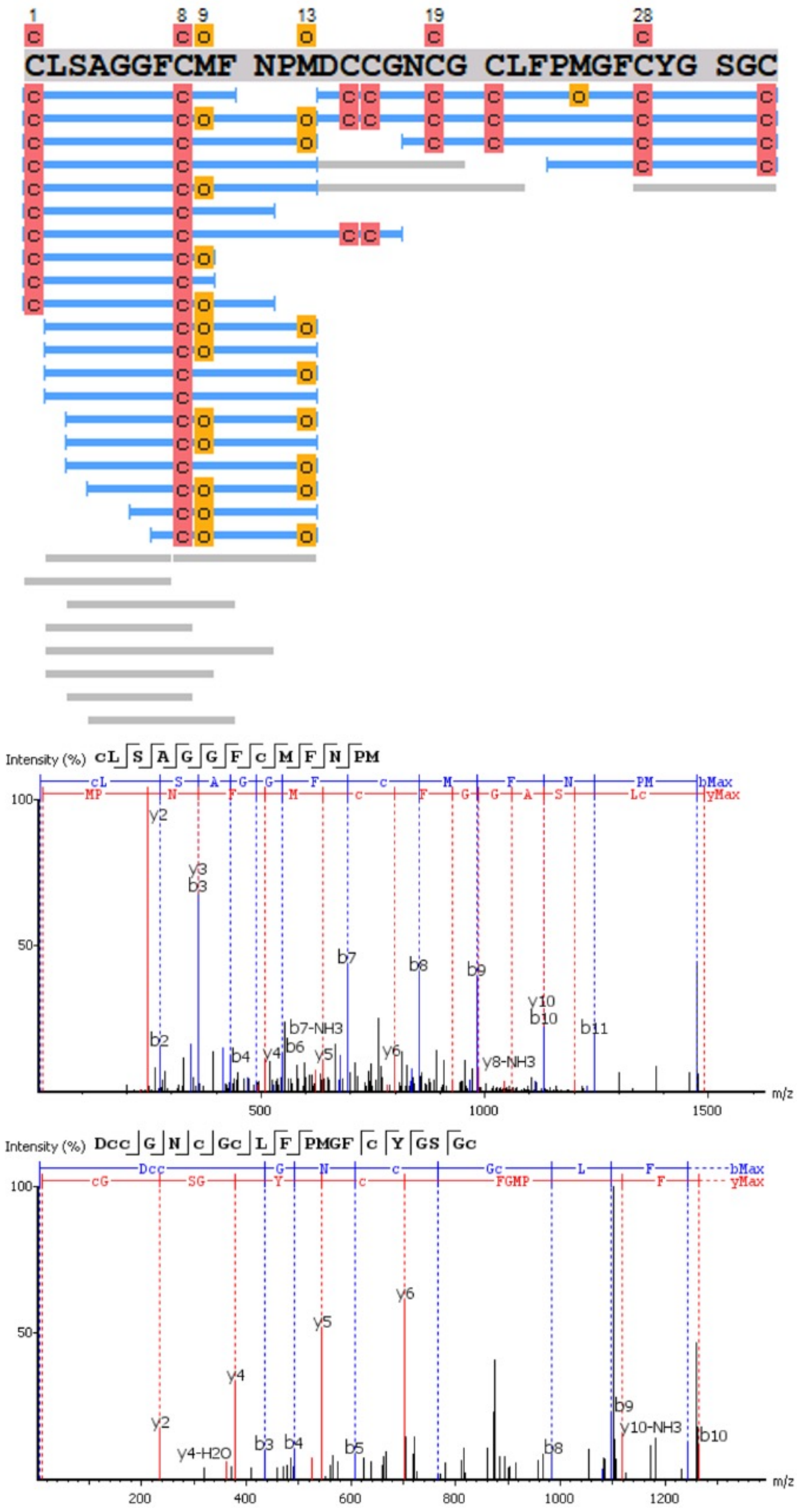

Figure S3. Peptide sequencing of cocotide tC2 using tims-TOF MS/MS

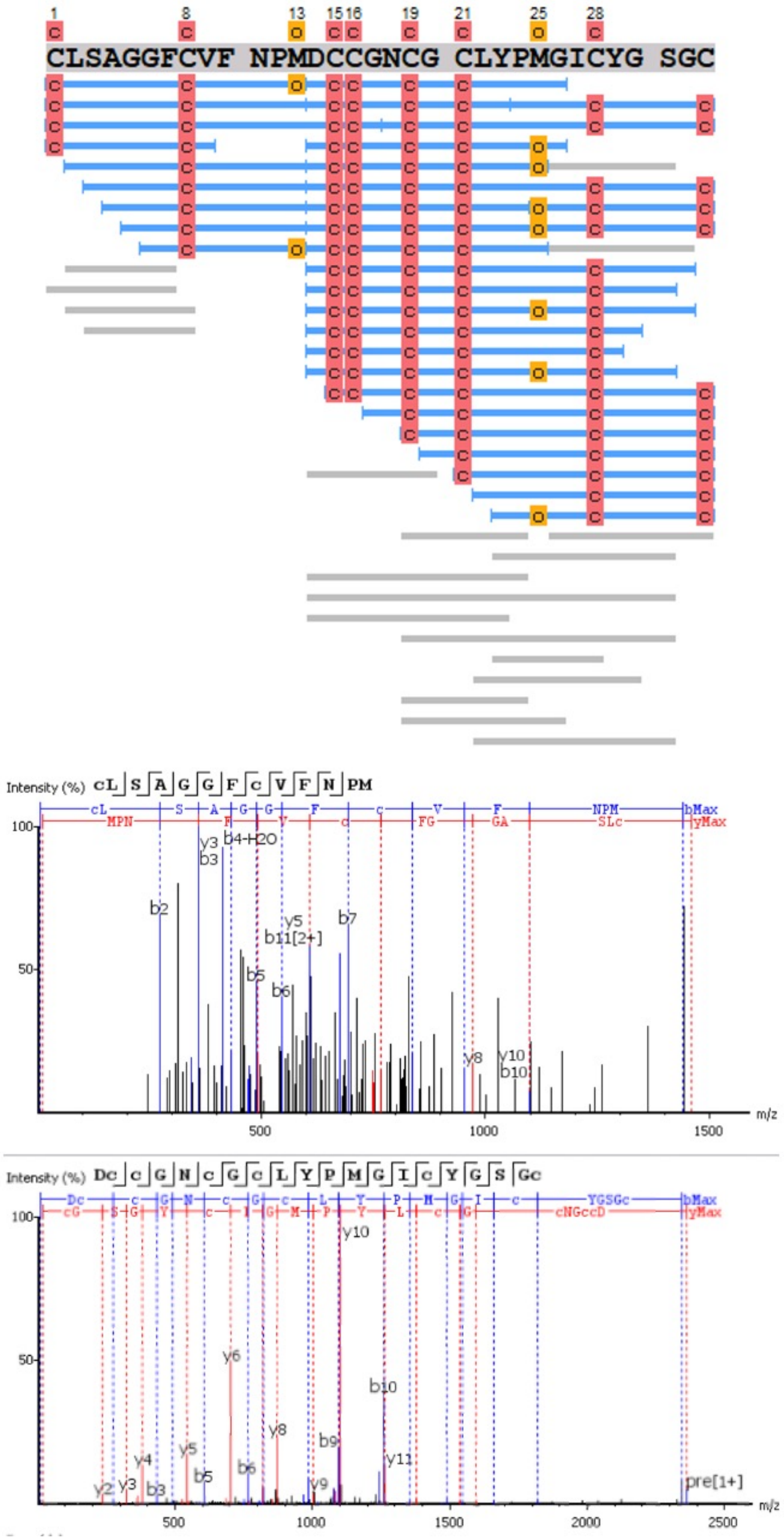

Figure S4. Peptide sequencing of cocotide tC7 using tims-TOF MS/MS

A
B
C

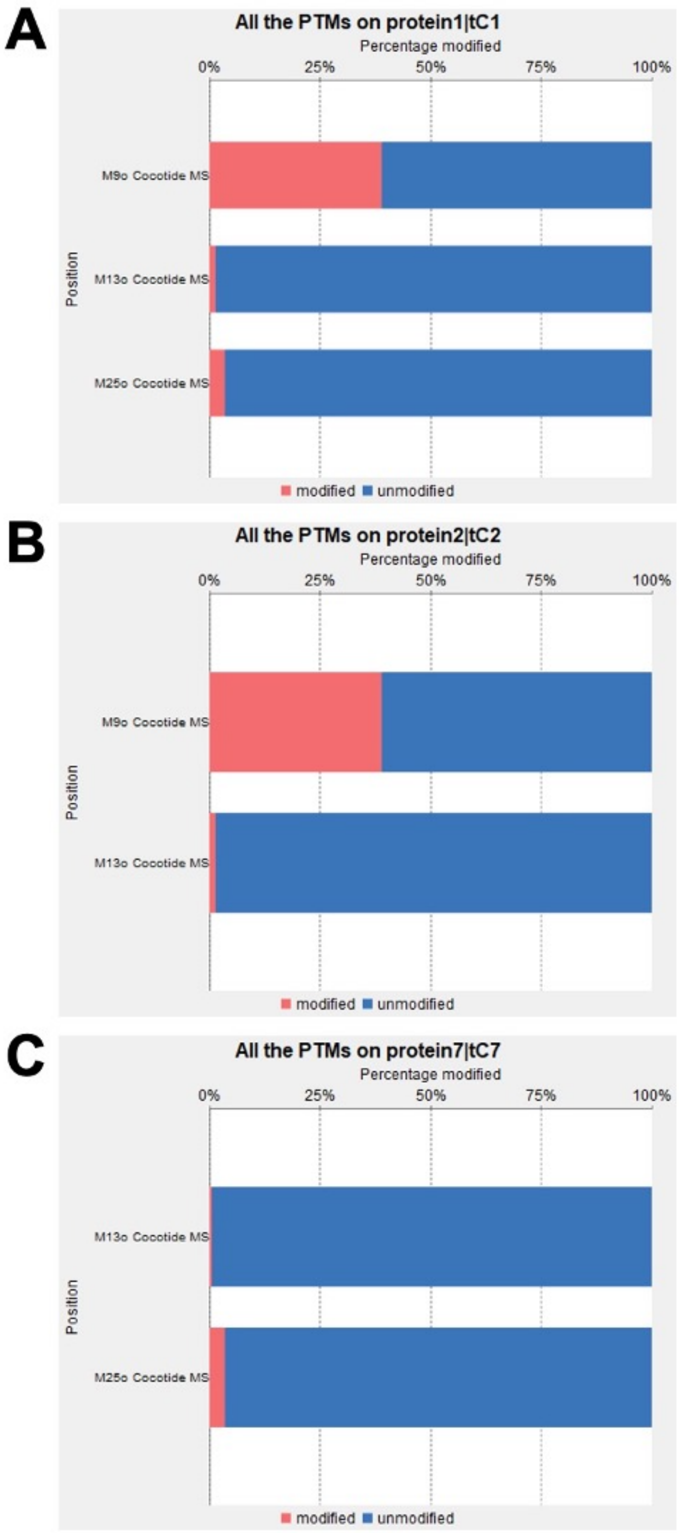

Figure S5. Percentage of methionine modification for cocotides tC1, tC2, and tC7.

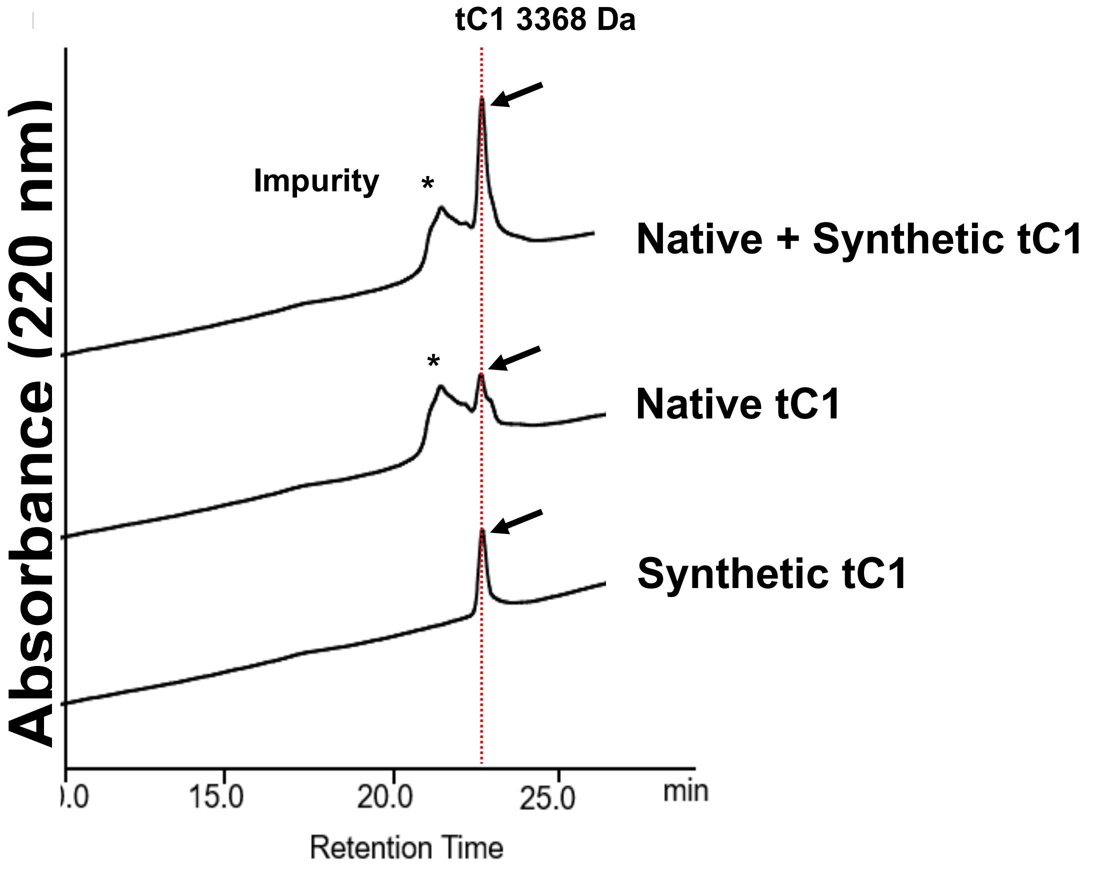

Figure S6. Co-elution of native and synthetic cocotide tC1 using reversed-phase high performance liquid chromatography. *denotes the impurity present.

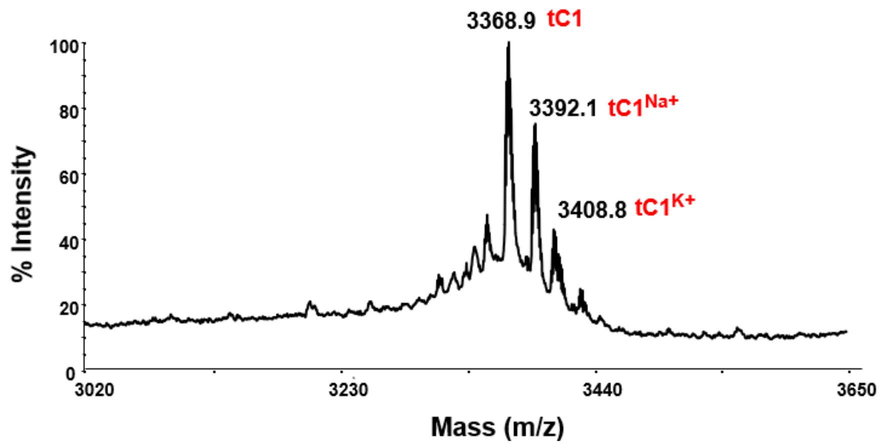

Figure S7. Mass spectrometry profile of synthetic cocotide tC1.

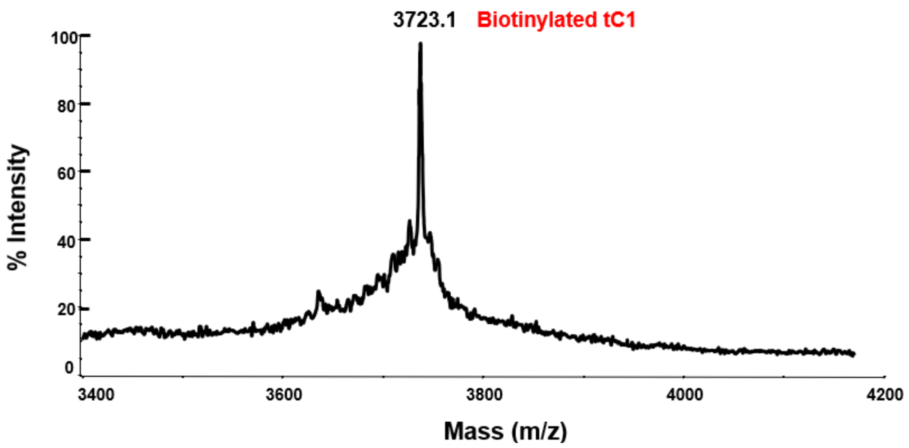

Figure S8. Mass spectrometry profile of Biotin-tC1.

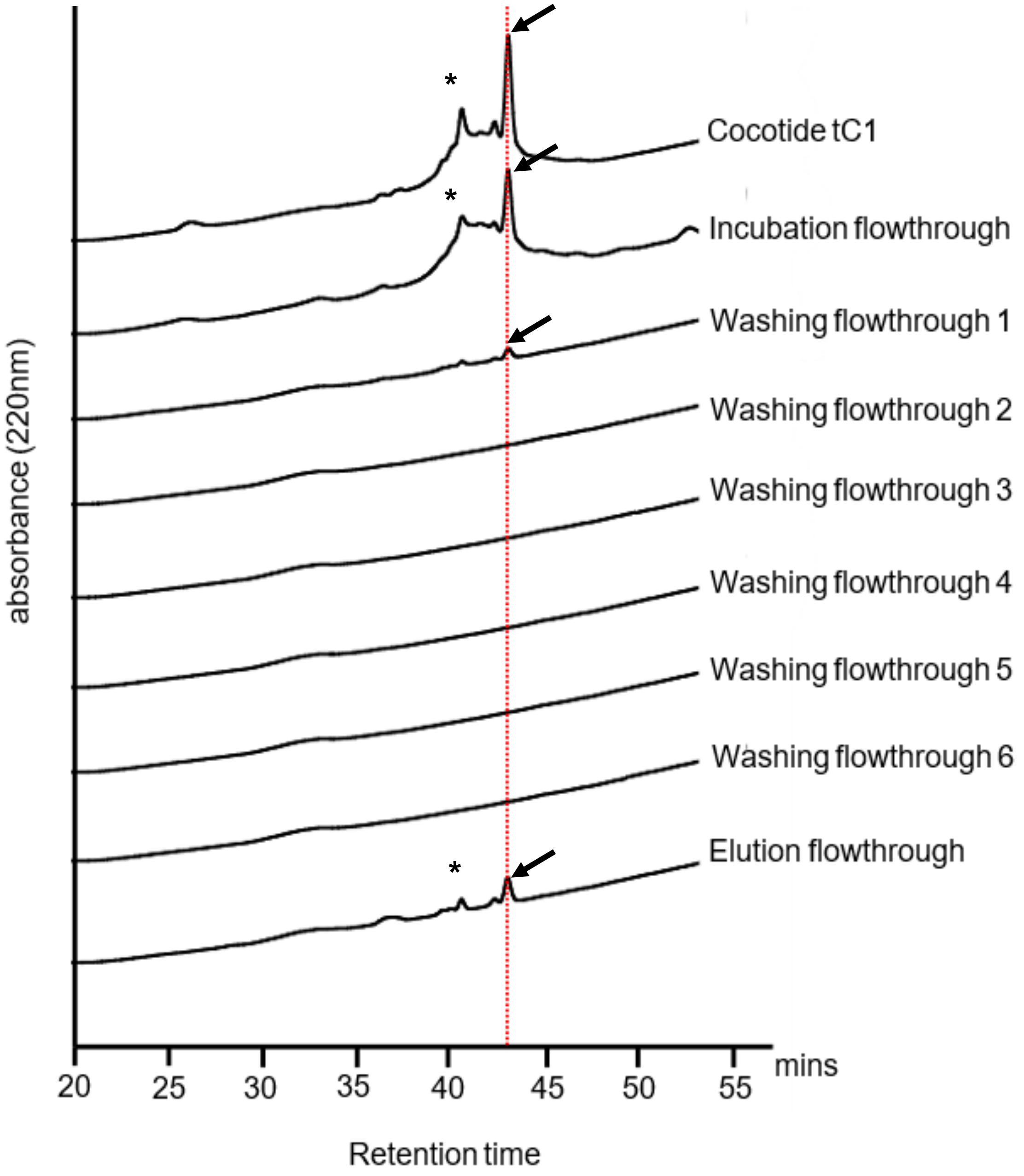

Figure S9. Iron-binding properties of cocotide tC1 *denotes the impurity present.
